# NEDD4-1: from molecular mechanisms to clinical translation in human diseases

**DOI:** 10.3389/fphar.2026.1897001

**Published:** 2026-10-12

**Authors:** Haifen Tian, Yongsheng Huang, Yun Liang, Chengjun Huang, Bingbing Wei, Chang-Neng Liao, Weijie Zhou

**Affiliations:** 1 Department of Pharmacy, Baise People’s Hospital, Affiliated Southwest Hospital of Youjiang Medical University for Nationalities, Baise, China; 2 Department of Neurosurgery, Baise People’s Hospital, Affiliated Southwest Hospital of Youjiang Medical University for Nationalities, Baise, China; 3 Department of Neurology, Baise People’s Hospital, Affiliated Southwest Hospital of Youjiang Medical University for Nationalities, Baise, China; 4 Department of Laboratory, Baise People’s Hospital, Affiliated Southwest Hospital of Youjiang Medical University for Nationalities, Baise, China

**Keywords:** cancer, E3 ubiquitin ligase, Nedd4-1, PTEN, ubiquitin ligase

## Abstract

Neural precursor cell expressed developmentally downregulated protein 4-1 (NEDD4-1), the founding member of the HECT family of E3 ubiquitin ligases, mediates substrate protein ubiquitination through its C2, WW, and HECT domains. It plays a key role in protein degradation via both the ubiquitin-proteasome system (UPS) and the lysosomal pathway. Recent evidence highlights the complexity of NEDD4-1’s functions. This review synthesizes advancements in the regulatory mechanisms of NEDD4-1, emphasizing its critical involvement in cancer initiation and progression. Beyond oncology, it highlights NEDD4-1’s vital functions in maintaining nervous system integrity, metabolic balance, cardiovascular health, and immune regulation. By examining the structural features, physiological roles, expression profiles in various diseases, molecular regulatory networks, and potential clinical applications of NEDD4-1, this review provides a comprehensive framework for understanding its biological mechanisms and advancing targeted therapeutic strategies.

## 1 Introduction

Ubiquitination, a critical post-translational modification, regulates protein stability, localization, and function through a cascade involving ubiquitin-activating enzymes (E1), ubiquitin-conjugating enzymes (E2), and ubiquitin ligases (E3). It plays a role in virtually all cellular processes, with E3 ligases determining substrate specificity (Rotin and Prag, 2024). HECT family E3 ligases transfer ubiquitin via their characteristic HECT domain, and NEDD4-1 (also known as Nedd4), the founding member of this family, has emerged as a central regulator of both physiological processes and disease progression since its identification. NEDD4-1 features a canonical domain structure: an N-terminal C2 domain that mediates membrane localization, four WW domains that recognize substrates (typically binding the PPXY motif), and a C-terminal HECT catalytic domain (Rotin and Prag, 2024).

Initially, NEDD4-1 was considered a prototypical oncoprotein due to its role in mediating the ubiquitination and degradation of the tumor suppressor PTEN (Alrosan et al., 2025; Huang et al., 2019). However, advances in proteomics, molecular biology, and animal models have expanded the range of NEDD4-1 substrates. Its functions are now understood to be highly pleiotropic and context-dependent, influenced by cell type, upstream signaling pathways, post-translational modifications, and the expression levels of specific isoforms or adaptor proteins (Kefalas et al., 2025; Kefalas and Rotin, 2023; Jiang et al., 2019). This review aims to consolidate these complexities, offering a comprehensive overview of NEDD4-1’s regulatory mechanisms and diverse roles in physiology and disease, thus providing a broad perspective for further research in this field.

## 2 Structural features of NEDD4-1

NEDD4-1 exhibits the hallmark structural features of HECT family E3 ubiquitin ligases. Its N-terminal C2 domain is responsible for calcium-dependent membrane localization, mediating both the association of NEDD4-1 with the cell membrane and its autoinhibition (Huang et al., 2019; Kefalas et al., 2025; Wang et al., 2010). The four central WW domains (WW1-WW4) form the core substrate-recognition module, with WW3 showing the strongest affinity for PY motifs. This domain specifically recognizes substrates through interactions with proline-rich regions (Huang et al., 2019; Kefalas et al., 2025; Bobby et al., 2013; Schulte et al., 2018; Panwalkar et al., 2016a; Panwalkar et al., 2016b). The C-terminal HECT domain is responsible for receiving and transferring ubiquitin molecules, catalyzing either mono-ubiquitination or polyubiquitination of target substrates (Huang et al., 2019; Kefalas et al., 2025; Persaud et al., 2011). Notably, NEDD4-1 also encodes a primate-specific splice isoform, Nedd4-1(NE), whose extended N-terminal segment partially substitutes for the C2 domain. This isoform, localized to late endosomes, regulates autophagy, highlighting a regulatory mechanism where NEDD4-1 achieves functional diversity through alternative splicing (Figure 1) (Kefalas et al., 2025; Kefalas and Rotin, 2023).

**FIGURE 1:**
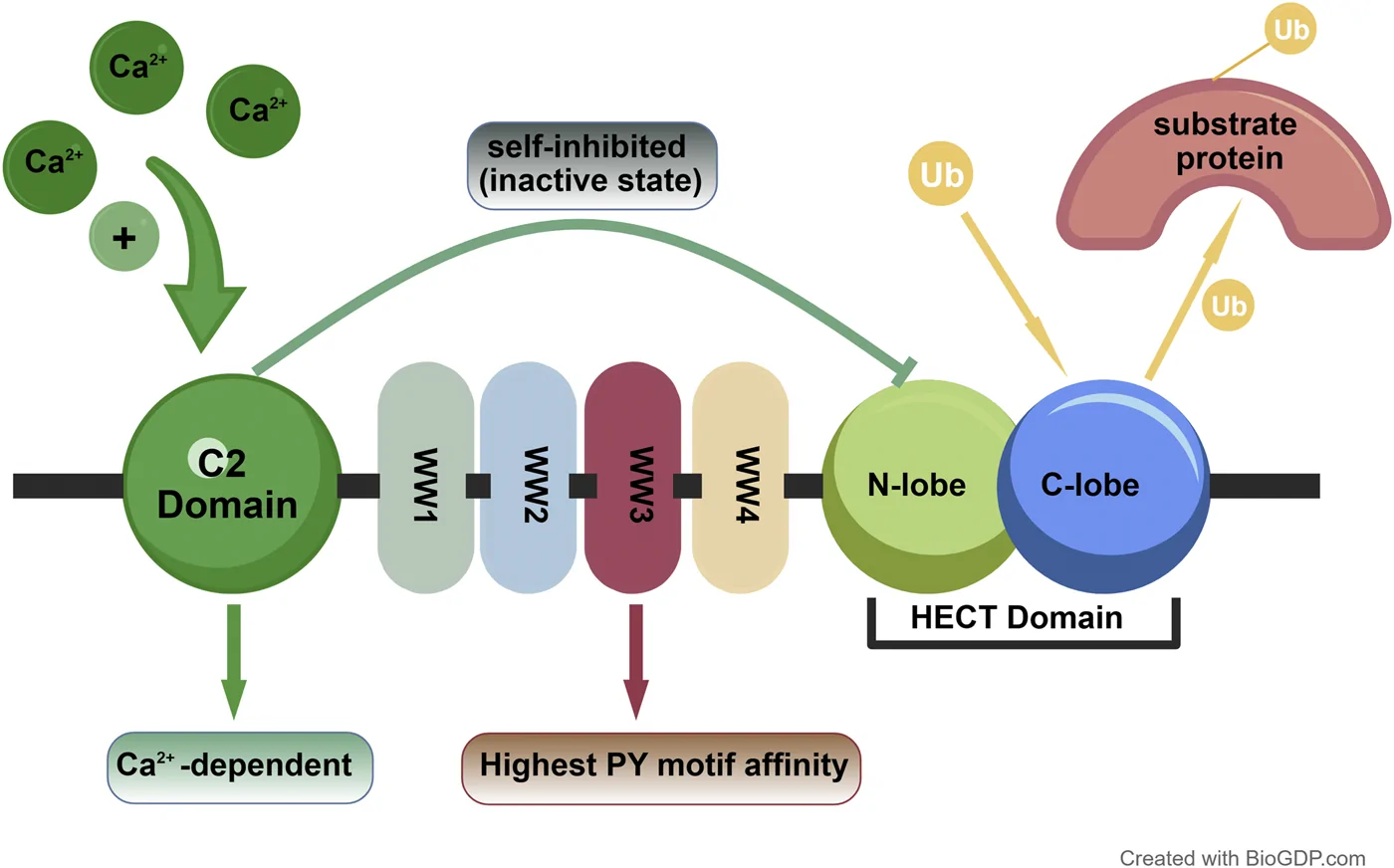
Schematic diagram of NEDD4-1 domain organization. NEDD4-1 comprises C2, WW, HECT, and other domains. The C2 domain mediates calcium-dependent membrane localization: under low calcium conditions, it binds to the HECT domain, forming an autoinhibited conformation; under high calcium conditions, it binds to cell membranes (e.g., plasma membrane or endosomal membranes), releasing the autoinhibition. The WW domain consists of four subdomains: WW1, WW2, WW3, and WW4. Among these, WW3 shows the highest affinity for the PY motif, enabling specific recognition via interactions with proline-rich regions in substrate proteins. The HECT domain is responsible for receiving and transferring ubiquitin molecules, catalyzing either mono-ubiquitination or polyubiquitination of substrate proteins.

## 3 Physiological functions of NEDD4-1

### 3.1 Regulation of protein stability

NEDD4-1 regulates the degradation of substrate proteins through the ubiquitination pathway, directing them to either the proteasomal or lysosomal degradation routes to maintain intracellular protein homeostasis (Figure 2A) (Jiang et al., 2019). For instance, NEDD4-1 ubiquitinates and promotes the degradation of voltage-gated calcium channels (VGCCs), thereby regulating calcium homeostasis and neuronal excitability (Liu et al., 2025). It also ubiquitinates the sodium-dependent vitamin C transporter-2 (hSVCT2), which in turn controls vitamin C uptake in neurons and epithelial cells (Teafatiller et al., 2023). Regarding the lysosomal pathway, the degradation mechanism operates as follows: Lysosomal-associated transmembrane protein 4α (LAPTM4α) recruits NEDD4-1 via its carboxy-terminal PY motif. Following ubiquitination, LAPTM4α is endocytosed into the lysosomal lumen through the endosomal sorting complexes required for transport (ESCRT)-mediated pathway, leading to its degradation (Zhang et al., 2021; Hirota et al., 2021).

**FIGURE 2:**
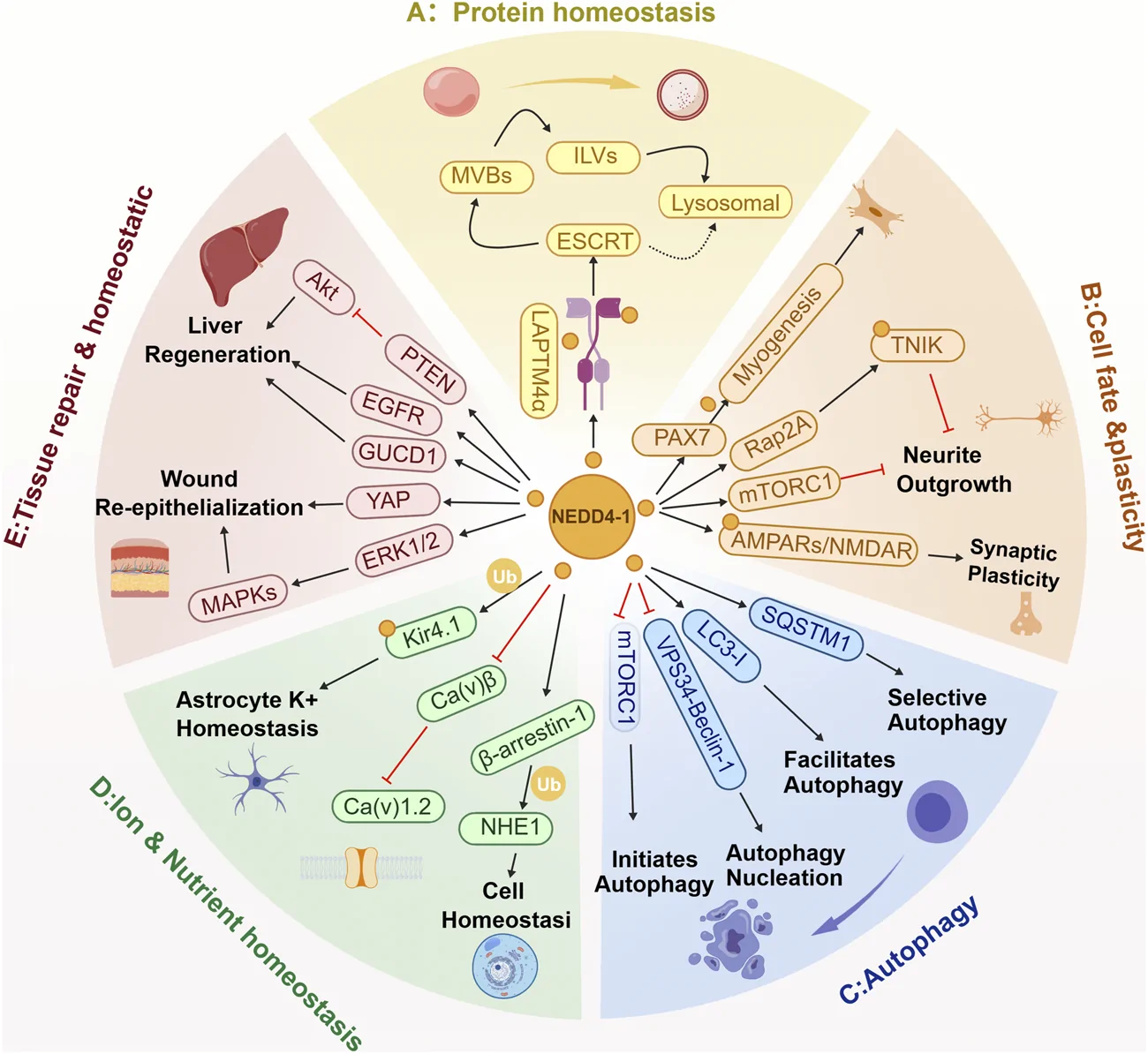
Physiological functions of NEDD4-1. These functions are categorized into five interconnected modules, with the core mechanism being NEDD4-1’s action through the ubiquitination of various substrates, leading to their degradation, altered trafficking, or functional modification. **(A)** NEDD4-1 regulates protein turnover via the proteasome and lysosomal (ESCRT-dependent) pathways. **(B)** NEDD4-1 regulates cell proliferation, differentiation (e.g., in muscle and neurons), morphogenesis, and synaptic plasticity. **(C)** NEDD4-1 promotes autophagosome formation, stabilizes autophagosomes, and extends multiple steps of autophagy and selective autophagy. **(D)** NEDD4-1 maintains ion and nutrient homeostasis by regulating the stability and membrane expression of various channels and transporters. **(E)** NEDD4-1 is essential for tissue repair and organ regeneration, particularly in skin wound healing and liver regeneration.

### 3.2 Cell proliferation and differentiation

In skeletal muscle regeneration, NEDD4-1 promotes the proliferation and differentiation of muscle progenitor cells by mediating the degradation of the transcription factor PAX7, which is critical for sustaining normal muscle regeneration (Cabezas et al., 2023). Beyond myogenesis, NEDD4-1 influences neuronal development: it drives neurite outgrowth and branching by modulating the Rap2A/TNIK signaling pathway (Kawabe et al., 2010; Drinjakovic et al., 2010; Kawabe and Brose, 2010), and is involved in mTORC1-dependent translational regulation, affecting neurite outgrowth in central nervous system neurons (Hsia and Kawabe, 2015; Hsia et al., 2014). Moreover, NEDD4-1 regulates synaptic strength by mediating the ubiquitination of α-amino-3-hydroxy-5-methyl-4-isoxazolepropionic acid receptors (AMPARs) and N-methyl-D-aspartate receptors (NMDARs), as well as their expression levels on the cell surface (Scudder et al., 2014). Additionally, NEDD4-1 is essential for cardiac development; conditional knockout of Nedd4-1 *in vivo* results in significant cardiac malformations, including double outlet right ventricle and atrioventricular cushion defects, alongside vascular developmental anomalies (Figure 2B) (Fouladkou et al., 2010; Wiszniak et al., 2026).

### 3.3 Autophagy and metabolic regulation

The Nedd4-1 isoform NE negatively regulates autophagy by interacting with the vesicle sorting protein VPS13B, inhibiting the maturation of late endosomes into autophagosomes (Kefalas et al., 2025). In contrast, canonical NEDD4-1 positively modulates autophagy at several stages: it initiates autophagy by suppressing mTORC1 (Li et al., 2015); enhances autophagy nucleation through stabilizing the VPS34-Beclin-1 complex (Xie et al., 2020a; Sun A. et al., 2017; Pei et al., 2017; Huang et al., 2020); facilitates autophagy elongation via interaction with LC3-I (Li et al., 2015; Sun A. et al., 2017; Behrends et al., 2010); and enables selective autophagy by stabilizing the autophagy receptor SQSTM1 (Figure 2C) (Sun A. et al., 2017; Lin et al., 2017). This dual regulatory mechanism of NEDD4-1 is presumably dependent on isoform specificity and dynamic changes in the cellular microenvironment.

### 3.4 Regulation of ion channels and transporters

NEDD4-1 is involved in the homeostatic regulation of various ion channels by modulating their membrane expression through ubiquitination (Figure 2D) (Teafatiller et al., 2023). For instance, it ubiquitinates the inwardly rectifying potassium channel Kir4.1, thereby regulating astrocyte membrane ion permeability and neuronal network activity (Altas et al., 2024). NEDD4-1 also regulates sodium reabsorption and fluid homeostasis by ubiquitinating the epithelial sodium channel (ENaC) (Zhong et al., 2014; Zhong and Liu, 2009; Henry et al., 2003). Additionally, it inhibits the trafficking of Ca(v)1.2 calcium channels to the plasma membrane during the early secretory pathway by interfering with the endoplasmic reticulum export pathway mediated by the Ca(v)β subunit (Rougier et al., 2011). Furthermore, NEDD4-1, in synergy with β-inhibin-1, mediates the ubiquitination of the sodium-hydrogen exchanger 1 (NHE1), contributing to the regulation of various biological processes, including intracellular pH, cell volume homeostasis, cell adhesion, migration, proliferation, and mechanosensing (Dai et al., 2010).

### 3.5 Tissue repair and homeostatic maintenance

NEDD4-1 also plays a pivotal role in epidermal homeostasis and wound repair, facilitating wound re-epithelialization through modulation of the ERK1/2/MAPK and YAP signaling pathways in keratinocytes (Figure 2E) (Yan et al., 2022; Ye et al., 2026). In liver regeneration models, NEDD4-1 is pivotal in post-hepatectomy liver regeneration by regulating growth factor receptor internalization and downstream mitogenic signaling activation, or by modulating GUCD1 protein function during hepatocyte proliferation (Bachofner et al., 2017; Bellet et al., 2014). Furthermore, the hepatotropic growth-promoting effects of the constitutive androstane receptor (CAR) depend on FoxM1-Nedd4-mediated PTEN suppression and subsequent Akt pathway activation (Bjij et al., 2018).

## 4 Molecular regulatory mechanisms of NEDD4-1

### 4.1 Self-inhibition and allosteric activation

Crystal structure analyses reveal that the C2 domain of NEDD4-1 directly interacts with its HECT domain, acting as a “molecular arm” that sequesters the catalytic center, thereby keeping NEDD4-1 in a resting state (Jiang et al., 2019). This conformation not only hinders efficient binding to E2 ubiquitin-conjugating enzymes but also restricts substrate access. Autoinhibition is relieved upon binding of adaptor proteins, such as NDFIP1/2, which interact with NEDD4-1’s WW domain via their PY motifs (Figure 3A). These proteins also anchor NEDD4-1 to specific membrane compartments (e.g., endosomal membranes) through their transmembrane properties. This interaction induces a conformational change in NEDD4-1, leading to dissociation of the C2 domain and release of autoinhibition. Simultaneously, it recruits substrates, like PTEN, to the catalytic center, facilitating “spatially localized ubiquitination” (Jiang et al., 2019; Gorla et al., 2022; Putz et al., 2012). Dysregulated NDFIP1 expression in neurological disorders disrupts NEDD4-1’s substrate-specific regulation, particularly towards synaptic receptors, contributing to synaptic dysfunction (Xing et al., 2016; Lackovic et al., 2012).

**FIGURE 3:**
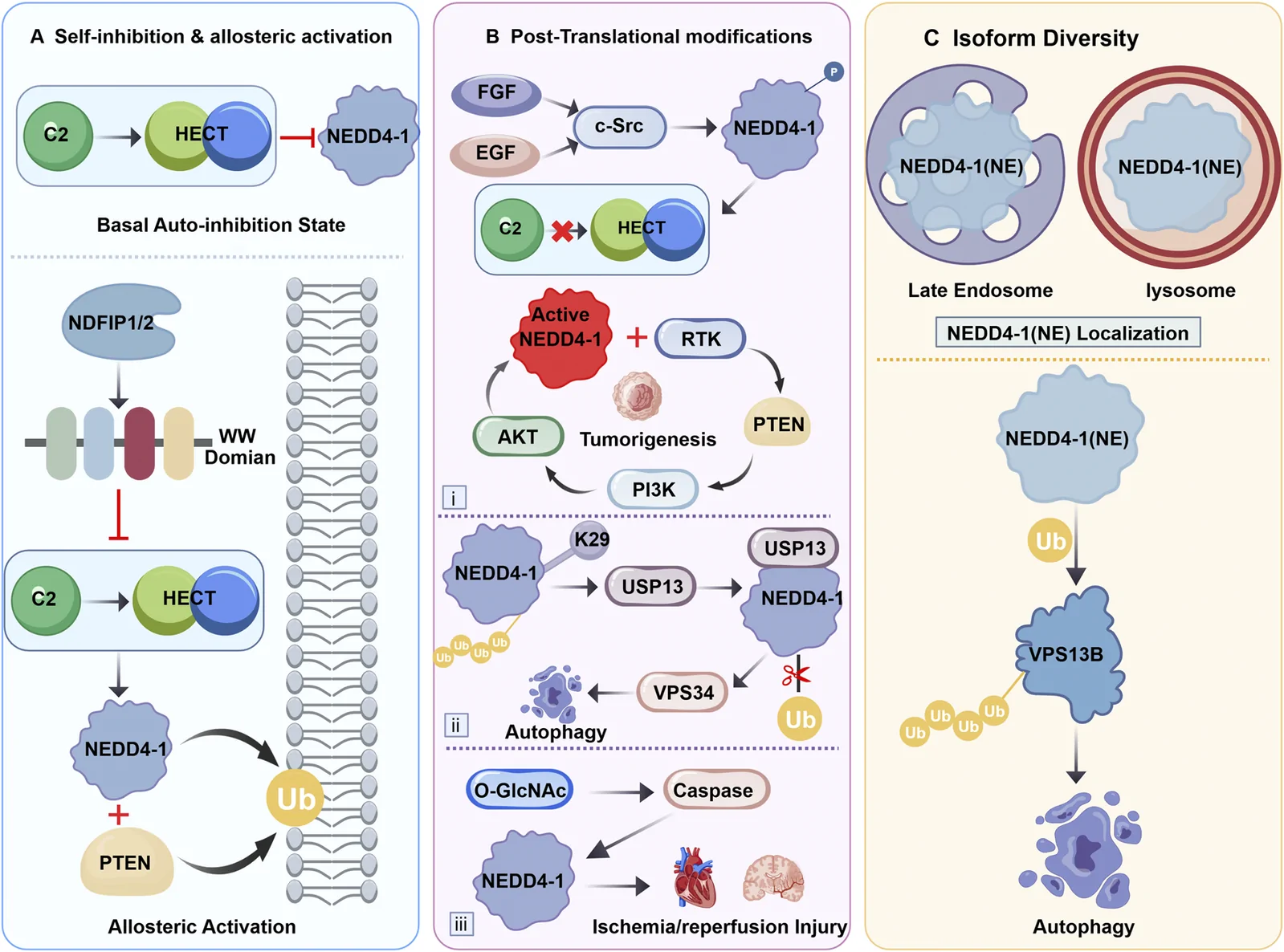
Multilevel mechanisms regulating NEDD4-1 function. **(A)** Autostivation and Allosteric Activation: Under basal conditions, the C2 domain interacts with the HECT domain, maintaining NEDD4-1 in an autostivated conformation. NDFIP1/2 proteins recruit NEDD4-1 to membranes via their PY motif binding to the WW domain, inducing a conformational change that releases autostivation and enables efficient substrate ubiquitination. **(B)** Post-Translational Modifications: (i) Phosphorylation of Y31 and Y556 by growth factor-activated kinases (e.g., c-Src) enhances catalytic activity and disrupts autoinhibition. The NEDD4-1(NE) isoform undergoes serine/threonine phosphorylation, forming a 14-3-3 binding site that may sequester the protein in the cytoplasm. (ii) NEDD4-1 K29-linked auto-ubiquitination recruits USP13 to stabilize VPS34 and promote autophagy. (iii) O-glycosylation modulates NEDD4-1 stability via a cysteine protease-mediated pathway, linking cellular metabolism to protein homeostasis. **(C)** Isoform Diversity: The primate-specific NEDD4-1(NE) isoform localizes to late endosomes/lysosomes and regulates autophagy via VPS13B-mediated ubiquitination, representing an independent signaling axis responsive to amino acid signaling.

### 4.2 Post-translation refinement

#### 4.2.1 Phosphorylation

Growth factor signaling pathways, including FGF and EGF, activate protein kinases like c-Src, which phosphorylate NEDD4-1 at key tyrosine residues (Y31 and Y556). This phosphorylation not only enhances the catalytic activity of the HECT domain but also disrupts the intramolecular interactions between the C2 and HECT domains, leading to sustained activation of NEDD4-1 in response to signaling (Persaud et al., 2014). In tumorigenesis, this mechanism forms a positive feedback loop: receptor tyrosine kinase (RTK) signaling activates NEDD4-1, which then mediates the ubiquitin-dependent degradation of tumor suppressors such as PTEN. This degradation promotes pro-survival signaling pathways like PI3K/AKT, which in turn sustains NEDD4-1’s active state. In contrast, the primate-specific NEDD4-1 isoform (NEDD4-1(NE)) undergoes serine/threonine phosphorylation in its N-terminal extension, creating a binding motif for 14-3–3 proteins. The interaction between 14-3-3 and NEDD4-1(NE) sequesters it in the cytoplasm through a “molecular sequestration” mechanism, or impairs its binding to specific substrates (Shao et al., 2017; Wang et al., 2007; Ahn et al., 2008; Garcia-Cao et al., 2012; Li et al., 2014; Huang et al., 2017). This allows NEDD4-1(NE) to respond to nutrient-sensing pathways independently of mTORC1 (Kefalas and Rotin, 2023). These mechanisms offer valuable insights into NEDD4-1’s role in metabolic disorders and cancer-associated metabolic reprogramming (Figure 3B).

#### 4.2.2 Ubiquitination

Additionally, the K29-linked auto-ubiquitination of NEDD4-1 functions not only as a degradation signal but also as a “recruitment motif” for deubiquitinating enzymes like USP13. This recruitment facilitates the formation of the NEDD4-1-USP13 complex, which deubiquitinates and stabilizes autophagy-related proteins such as VPS34 (Xie et al., 2020a; Huang et al., 2020; Xie et al., 2020b). These findings position NEDD4-1 as a key regulatory node in the autophagy pathway, with its dysregulation directly linked to impaired protein aggregate clearance in neurodegenerative diseases and aberrant survival adaptations in tumor cells.

#### 4.2.3 O-GlcNAc glycosylation

The ubiquitin ligase NEDD4-1 undergoes O-GlcNAc glycosylation (Zaro et al., 2011), and O-GlcNAc levels indirectly regulate its stability via a caspase-mediated pathway (Jiang et al., 2016). The identification of the O-GlcNAc-caspase-NEDD4-1 regulatory axis challenges the traditional boundaries between distinct cellular signaling pathways, integrating cellular metabolic status (via O-GlcNAc glycosylation), programmed cell death signaling (caspase), and a central regulator of protein homeostasis (NEDD4-1). This axis offers a novel framework for understanding the molecular mechanisms of protein homeostasis dysregulation in metabolic stress-associated diseases. Ischemia/reperfusion injury, seen in conditions such as myocardial infarction and stroke, represents a paradigm of this pathology, marked by profound metabolic perturbations, oxidative stress, fluctuations in O-GlcNAc levels, and caspase activation. Given NEDD4-1’s known cardioprotective effects (Hu et al., 2017), its degradation triggered by elevated O-GlcNAc levels during early ischemia may exacerbate tissue damage, highlighting a potential therapeutic window for targeting this regulatory node with clinical implications.

#### 4.2.4 Diversity of isomers

The discovery of the primate-specific NEDD4-1(NE) isoform introduces a new layer of complexity in NEDD4-1-mediated signaling. Due to its distinct subcellular localization to late endosomes/lysosomes and its elevated basal activity, NEDD4-1(NE) likely represents a signaling axis independent of the classical NEDD4-1 isoform. This isoform responds to amino acid signals and modulates endosomal maturation and autophagic flux by ubiquitinating factors such as VPS13B (Figure 3C) (Kefalas et al., 2025; Kefalas and Rotin, 2023). Dysregulation of NEDD4-1(NE) could contribute to lysosomal storage disorders and autophagy-defective cancers, presenting an overlooked mechanism in these pathologies.

## 5 Role of NEDD4-1 in tumors

### 5.1 Glioma

Glioblastoma (GBM) is the most common and aggressive primary malignant tumor of the central nervous system. Although temozolomide (TMZ) remains the standard treatment, the development of TMZ resistance poses a significant challenge to clinical efficacy. Accumulating evidence shows that the E3 ubiquitin ligase NEDD4-1 is markedly overexpressed in both GBM tissues and TMZ-resistant cell lines (Chuang et al., 2021). The molecular mechanisms underlying NEDD4-1-mediated TMZ resistance primarily involve three key aspects: First, NEDD4-1 directly mediates the ubiquitination of PTEN, leading to its proteasomal degradation. This degradation alleviates PTEN-dependent inhibition of the PI3K/AKT/mTOR signaling pathway, thereby promoting tumor cell survival and proliferation (Chuang et al., 2021; Zhang et al., 2013). Second, the loss of PTEN function activates AKT, which subsequently phosphorylates and stabilizes the transcription factor NRF2. Stabilized NRF2 upregulates its downstream effector, heme oxygenase-1 (HO-1), enhancing the tumor cells’ ability to scavenge reactive oxygen species (ROS) and counteract TMZ-induced oxidative stress damage (Chuang et al., 2021). Third, epigenetic dysregulation contributes to NEDD4-1 upregulation in TMZ-resistant cells: abnormal downregulation of miR-3129–5p and miR-199b-3p increases NEDD4-1 mRNA stability, resulting in elevated protein expression (Chuang et al., 2021). Notably, combined treatment with the NEDD4-1 inhibitor indole-3-carbinol (I3C) or its derivatives and TMZ has been shown to effectively reverse TMZ resistance in both *in vitro* and *in vivo* models (Chuang et al., 2021; Aronchik et al., 2014; Quirit et al., 2017).

Furthermore, NEDD4-1 plays a pivotal role in regulating glioma cell migration and invasion, thereby facilitating tumor progression. The underlying mechanisms are well-documented: (1) Targeted degradation of CNrasGEF: NEDD4-1 directly ubiquitinates cyclic nucleotide-Ras guanine nucleotide exchange factor (CNrasGEF), promoting its proteasomal degradation. Reduced CNrasGEF expression correlates negatively with enhanced migration and invasion capacities of GBM cells (Wang et al., 2007). (2) K63-linked ubiquitination of Rap2a: NEDD4-1 mediates K63-linked ubiquitination of Ras-associated protein Rap2a, inhibiting the activity of its GTP-bound active form (GTP-Rap2a). This modification promotes the formation of a Rap2a complex with TRAF2 and NCK-interacting kinase (TNIK), ultimately driving cell migration and invasion (Wang et al., 2017). (3) Transcriptional regulation by FoxM1B: In GBM, the transcription factor FoxM1B is overexpressed and directly transactivates NEDD4-1. This upregulation accelerates PTEN degradation and activates the Akt signaling pathway, inducing the malignant transformation of immortalized astrocytes and recapitulating the pathological phenotype of GBM (Dai et al., 2010).

### 5.2 Lung cancer

NEDD4-1 is significantly upregulated in non-small cell lung cancer (NSCLC), particularly in its lung adenocarcinoma (ADC) subtype, and its elevated expression is strongly associated with poor clinical outcomes, lymph node metastasis, advanced TNM stage, and chemoresistance (Song et al., 2018; Amodio et al., 2010; Gao et al., 2025; Shao et al., 2018; Booth et al., 2018)^.^

In lung adenocarcinoma, high NEDD4-1 expression negatively correlates with PTEN levels and positively correlates with phosphorylated Akt (p-Akt) levels (Huang et al., 2017). Functional assays have shown that silencing NEDD4-1 inhibits the proliferation, migration, and invasion of lung adenocarcinoma cells (e.g., A549), enhances their chemosensitivity to cisplatin and paclitaxel, restores PTEN expression, and suppresses PI3K/Akt pathway activation (Song et al., 2018).

In NSCLC, NEDD4-1 is a key mediator of acquired resistance to epidermal growth factor receptor tyrosine kinase inhibitors (EGFR-TKIs, e.g., erlotinib) through PTEN degradation. In erlotinib-resistant HCC827/ER cells, NEDD4-1 is upregulated while PTEN protein levels decrease, and NEDD4-1 knockdown restores PTEN expression, partially reversing the drug resistance (Sun H. et al., 2017). Approximately 25% of NSCLC cases exhibit NEDD4-1 overexpression, driven by amplification of its 15q21 gene locus (Amodio et al., 2010). Importantly, the oncogenic potential of NEDD4-1 is not limited to PTEN-intact cells; its overexpression promotes tumor growth through alternative substrates, including Akt, even in PTEN-deficient contexts (Huang et al., 2020).

### 5.3 Gastrointestinal tumors

#### 5.3.1 Gastric cancer (GC)

In GC, NEDD4-1 is significantly upregulated and closely correlates with poor prognosis. Its oncogenic role is driven by multi-layered molecular regulatory mechanisms (Kim et al., 2008; Han et al., 2025). A proteomic analysis identified NEDD4-1 as a potential downstream effector of microtubule-associated serine/threonine kinase-like protein (MASTL) in driving GC progression, highlighting MASTL as a promising therapeutic target for this malignancy. Functional experiments showed that MASTL silencing not only abrogated the malignant phenotype of GC cells but also downregulated NEDD4-1 protein levels. Similarly, NEDD4-1 knockdown markedly impaired the migration, invasion, and proliferation of GC cells (Choi et al., 2024). Furthermore, tumor-associated macrophage (TAM)-derived exosomes (M2-exo) enriched in the long non-coding RNA CRNDE can be transferred into GC cells, promoting NEDD4-1-mediated ubiquitination and degradation of PTEN. This CRNDE-NEDD4-1-PTEN axis induces cisplatin resistance, while silencing CRNDE in M2-exo reverses the chemoresistant phenotype and inhibits both GC cell proliferation and xenograft growth (Xin et al., 2021). In gastric cardia adenocarcinoma (GCA), NEDD4-1 serves as a specific biomarker, with overexpression detected in 83% of clinical specimens and validated as an independent prognostic factor. Patients negative for NEDD4-1 exhibit a 5-year overall survival rate of 96%, while NEDD4-1 overexpression is significantly associated with advanced TNM stage and reduced overall survival. Functional assays further confirmed that NEDD4-1 knockdown drastically suppresses the migratory and invasive potential of GCA cells (Sun et al., 2014).

#### 5.3.2 Colorectal cancer (CRC)

The function of NEDD4-1 in colorectal cancer is tissue-specific and context-dependent. Studies in mouse models have shown that intestinal-specific knockout of Nedd4-1 does not initiate tumor formation but significantly accelerates intestinal tumor progression when combined with an Apc gene mutation (Apc^+^/min) (Lu et al., 2016). At the molecular level, NEDD4-1 deficiency leads to upregulation of upstream transcription factors Lymphocyte Enhancer Factor 1 (LEF1) and YY1 in the WNT signaling pathway. This upregulation derepresses the WNT pathway, promoting tumorigenesis. These findings suggest that within the intestinal microenvironment, NEDD4-1 may exert tumor-suppressive effects by negatively regulating the WNT signaling pathway, thereby limiting tumor growth.

### 5.4 Breast cancer

In breast cancer, the interaction between NEDD4-1 and PTEN is complex and remains controversial. Although NEDD4-1 has been confirmed as an E3 ubiquitin ligase that targets PTEN, large-scale cohort studies in breast cancer have failed to show a consistent negative correlation between NEDD4-1 and PTEN at either the protein or mRNA level. Instead, a positive correlation has been observed in some subsets of cases (Chen et al., 2016; van de Vijver et al., 2002; vanʼt Veer et al., 2002). These findings suggest that NEDD4-1 is unlikely to be the primary driver of PTEN downregulation in breast cancer. Its pro-tumorigenic role is more likely mediated through PTEN-independent molecular pathways. For instance, the oncoprotein p34 (also known as SEI-1) can induce upregulation of NEDD4-1 at both the transcriptional and translational levels, thereby modulating the subcellular localization and protein stability of PTEN to activate the PI3K/AKT signaling pathway. However, the specific molecular mechanisms underlying this regulatory axis and its clinical implications still require further investigation (Jung et al., 2013; Shrestha et al., 2017; Hong et al., 2014).

### 5.5 Melanoma

In melanoma, NEDD4-1 is highly expressed and represents a promising therapeutic target. Its core oncogenic mechanism involves the regulation of PTEN protein stability (Aronchik et al., 2014; Cheng et al., 2021). In melanoma cells with wild-type PTEN, the natural compound I3C and its synthetic derivatives bind directly to NEDD4-1 and inhibit its ubiquitin ligase activity. This prevents PTEN ubiquitination and subsequent degradation, thereby stabilizing PTEN protein. Stabilized PTEN induces G1-phase cell cycle arrest and apoptosis, resulting in a significant suppressive effect on tumor growth *in vivo*. Notably, I3C does not exhibit noticeable biological effects in melanoma cells with PTEN mutation or deletion (Ahn et al., 2008). Further medicinal chemistry studies have shown that structural analogues of I3C, such as compound 2242, have enhanced bioactivity, with IC_50_ values for inhibiting NEDD4-1 ubiquitin ligase activity in the micromolar range (Garcia-Cao et al., 2012). The antiproliferative activity of these analogues correlates positively with their potency in inhibiting NEDD4-1, suggesting their potential as lead candidates for the development of NEDD4-1-targeted small-molecule inhibitors (Quirit et al., 2017).

### 5.6 Other tumors

#### 5.6.1 Pituitary adenoma

NEDD4-1 expression is significantly upregulated in invasive pituitary adenoma tissues compared to non-invasive adenomas and normal pituitary tissues, with its expression negatively correlated with PTEN levels in a statistically significant manner. These findings suggest that NEDD4-1 may drive the invasive progression of pituitary adenomas by mediating the ubiquitination and subsequent degradation of PTEN (Zhang et al., 2017).

#### 5.6.2 Multiple myeloma (MM)

In contrast to most solid tumors, NEDD4-1 is characterized by low expression in patients with MM, and this pattern is strongly associated with poor prognosis (Huang et al., 2020). Functional assays demonstrate that NEDD4-1 knockdown confers resistance to the proteasome inhibitor bortezomib in MM cells, while overexpression of wild-type NEDD4-1—rather than its enzyme-inactive mutant—enhances drug sensitivity. Mechanistically, NEDD4-1 directly ubiquitinates and degrades p-Akt (pAkt-Ser473), thus negatively regulating the pro-survival Akt signaling pathway. Aberrant activation of the Akt pathway due to low NEDD4-1 expression renders MM cells more susceptible to Akt inhibitors, providing a potential strategy for combination targeted therapy.

#### 5.6.3 Nasopharyngeal carcinoma (NPC)

In cisplatin-resistant NPC cell lines, NEDD4-1 expression is upregulated and significantly correlated with the epithelial-mesenchymal transition (EMT) phenotype. Silencing NEDD4-1 partially reverses the EMT process, restores epithelial cell characteristics, and enhances cellular sensitivity to cisplatin, indicating that NEDD4-1 plays a key regulatory role in the chemoresistance mechanism (Feng et al., 2017; Fang et al., 2024).

#### 5.6.4 Hepatocellular carcinoma (HCC)

NEDD4-1 also contributes to the initiation and progression of HCC by modulating the stability of specific substrate proteins (Bellet et al., 2014; Huang et al., 2017; Zhou et al., 2023). Additionally, NEDD4-1 interacts with guanylate cyclase domain-containing protein 1 (GUCD1) and regulates its protein stability. GUCD1 is highly expressed in both proliferating hepatocytes and HCC tissues, implying that the NEDD4-1-GUCD1 signaling axis plays a critical role in hepatic proliferative abnormalities and HCC progression (Bellet et al., 2014). Similarly, NEDD4-1 can interact with craniofacial development protein 1 (CFDP1) and participate in PTEN regulation, thereby promoting the progression of hepatocellular carcinoma (Zhou et al., 2023). The central role of NEDD4-1 in tumorigenesis is summarized in Figure 4. Table 1 provides a summary of ZEB1’s role in the progression of different tumors.

**FIGURE 4:**
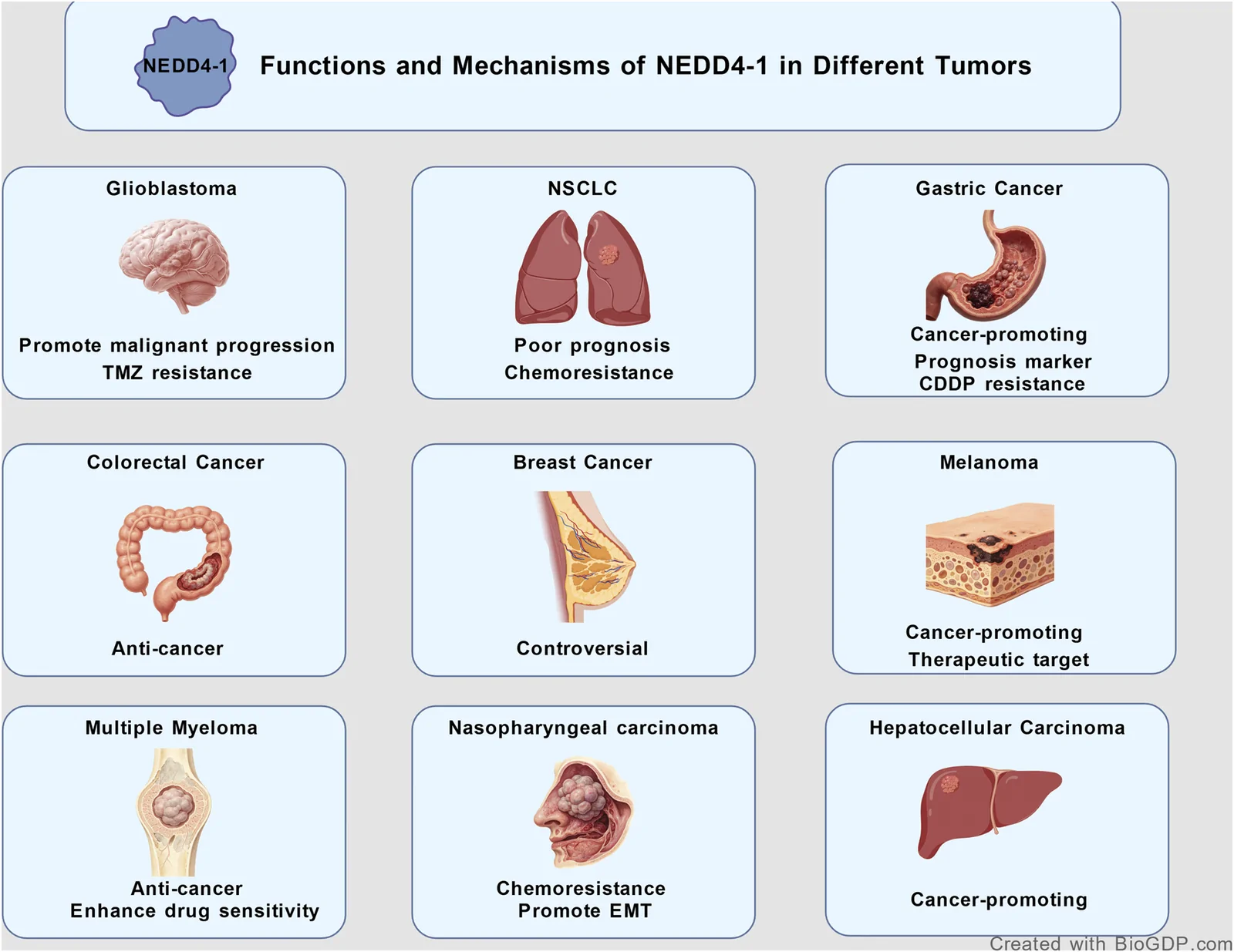
The central role of NEDD4-1 in tumor signaling networks. NEDD4-1 exerts tumor-promoting ef-fects in glioblastoma, non-small cell lung cancer (NSCLC), gastric cancer, melanoma, nasopharyngeal carcinoma and hepatocellular carcinoma, which accelerates malignant progression, mediates chemoresistance and epitheli-al-mesenchymal transition (EMT), and can serve as prognostic biomarker or therapeutic target. Conversely, NEDD4-1 acts as a tumor suppressor in colorectal cancer and multiple myeloma by improving drug sensitivity. The functional role of NEDD4-1 in breast cancer remains controversial. Created with BioGDP.

**TABLE 1:** Functions and Mechanisms of NEDD4-1 in Different Tumors.

Cancer	Function	Mechanism	References
Glioblastoma	PTEN degradation CNrasGEF ubiquitination Rap2a K63 ubiquitination	Mediates PTEN ubiquitination and degradation, releasing inhibition of the PI3K/AKT/mTOR pathway to promote cell survival and proliferation; activates AKT by degrading PTEN, stabilizes NRF2, and upregulates HO-1 to counteract TMZ-induced oxidative stress Ubiquitinates and degrades CNrasGEF, enhancing cell migration and invasion Modifies Rap2a to promote its complex formation with TNIK, driving cell migration	Poor prognosis (Chuang et al., 2021; Zhang et al., 2013; Wang et al., 2017)
Lung adenocarcinoma/non-small cell lung cancer	PTEN degradation	Mediates the ubiquitination degradation of PTEN, activates the PI3K/AKT pathway, and promotes proliferation, invasion, and resistance to chemotherapy (cisplatin, paclitaxel) and EGFR-TKI (erlotinib)	Poor prognosis (Song et al., 2018; Amodio et al., 2010; Gao et al., 2025)
Stomach cancer	PTEN degradation, exosomal lncRNA CRNDE regulation Stabilizing oncopro-proteins	Mediates the ubiquitination degradation of PTEN and activates the pro-survival pathway Tumor-associated macrophage-derived exosomes deliver CRNDE, promote NEDD4-1-mediated PTEN degradation, and induce cisplatin resistance Stabilizes proteins such as CNN1/PDLIM7 to exert pro-carcinogenic effects	Poor prognosis (Gorla et al., 2022; Persaud et al., 2014; Shao et al., 2018; Booth et al., 2018; Sun H et al., 2017)
Colorectal cancer	LEF1/YY1 degradation	NEDD4-1 inhibits tumorigenesis by targeting the degradation of transcription factors LEF1 and YY1, negatively regulating the WNT signaling pathway	Good prognosis (Lu et al., 2016)
Breast cancer	PTEN degradation HER3 degradation	As an E3 ligase for PTEN, but there is no consistent negative or even positive correlation with PTEN levels in large cohorts It may operate through non-PTEN-dependent mechanisms (e.g., regulation of PTEN subcellular localization) or transcriptional regulation by the oncoprotein p34 (SEI-1) It may mediate HER3 degradation and exert a tumor-suppressive effect	Controversial (Booth et al., 2018; Chen et al., 2016; Jung et al., 2013; Huang et al., 2015)
Melanoma	PTEN degradation	Degradation of PTEN by ubiquitination drives tumor growth	Poor prognosis (Bachofner et al., 2017)
Pituitary adenoma	PTEN degradation	Degrades PTEN and promotes aggressiveness	Poor prognosis (Zhang et al., 2017)
Multiple myeloma	pAkt-Ser473 degradation	Phosphorylated Akt (pAkt-Ser473) is degraded by ubiquitination to play a tumor inhibitory effect and enhance bortezomib sensitivity	Good prognosis (Huang et al., 2020)
Nasopharyngeal cancer	EMT regulation	Participates in EMT regulation to reduce cisplatin drug sensitivity	Poor prognosis (Feng et al., 2017)

## 6 NEDD4-1 and non-neoplastic diseases

### 6.1 Systemic diseases and neurodegenerative diseases

#### 6.1.1 Stress-related cognitive impairment

NEDD4-1 and the deubiquitinating enzyme USP8 form a rapid-responding equilibrium system that regulates the internalization and recycling of AMPA receptors (AMPARs) through ubiquitin modification and deubiquitination, respectively (Kawabe et al., 2010). Chronic stress upregulates NEDD4-1 via the glucocorticoid receptor-HDAC2-Ehmt2 epigenetic axis, resulting in excessive degradation of AMPARs in the prefrontal cortex. This mechanism serves as the structural basis for stress-related cognitive impairment (Rodrigues et al., 2016; Yuen et al., 2012).

#### 6.1.2 In hypoxic-ischemic encephalopathy (HIE)

In hypoxic-ischemic brain damage (HIBD) and oxygen-glucose deprivation (OGD) models, the PTEN protein undergoes monoubiquitination at lysine 13 (K13), a modification mediated by NEDD4-1. This post-translational modification is recognized as a key factor in driving neuronal injury. Notably, α- and γ-adaptin binding protein (Aagab) has been identified as a novel regulator of NEDD4-1, which enhances PTEN monoubiquitination by stabilizing or upregulating NEDD4-1 expression. This process suppresses PTEN nuclear translocation, contributing to the regulation of signaling pathway networks in neural injury (Dai et al., 2021).

#### 6.1.3 Alzheimer’s disease (AD)

In the hippocampus of patients with AD, NEDD4-1 is highly expressed (Teafatiller et al., 2023; Guntupalli et al., 2017). β-Amyloid (Aβ) oligomers recruit NEDD4-1 to synapses, where it mediates the ubiquitination-dependent degradation of the GluA1 subunit of AMPARs. This event contributes to synaptic dysfunction and subsequent cognitive impairment (Rodrigues et al., 2016; Guntupalli et al., 2017; Schwarz et al., 2010). Moreover, NEDD4-1 modifies P-glycoprotein (P-gp), a member of the ATP-binding cassette transporter family, via ubiquitination. This modification reduces P-gp membrane localization, impairs Aβ clearance across the blood-brain barrier, and exacerbates cerebral Aβ accumulation (Chai et al., 2022; Akkaya et al., 2015). PTEN, a key negative regulator of the PI3K/Akt pro-survival signaling pathway, is significantly downregulated in AD, mild cognitive impairment (MCI), and other neurodegenerative disorders, leading to aberrant Akt activation and highlighting its central role in neuropathological processes (Henry et al., 2003). Furthermore, studies have revealed that the nitric oxide (NO) signaling pathway integrates into this regulatory network by promoting NEDD4-1-mediated ubiquitination and degradation of PTEN through the ubiquitin-proteasome system (UPS) (Kwak et al., 2010). Nedd4 family interaction protein 1 (Ndfip1) also emerges as a novel candidate negative regulator of spatial memory formation. It interacts with NEDD4-1 to modulate the ubiquitination levels of Beclin 1 and PTEN in the hippocampus, contributing to the molecular regulation of AD-associated memory deficits (Hsu et al., 2023).

#### 6.1.4 Parkinson’s disease (PD)

Misfolded α-synuclein (α-Syn, aS) aggregation is a hallmark of PD and other neurodegenerative disorders (Won et al., 2022; Sugeno et al., 2014). Previous studies have shown a significant negative correlation between the activity of p21-activated kinase 4 (PAK4), a key negative regulator of aS aggregation, and the extent of aS aggregation in the brain tissues of patients with PD. NEDD4-1 interacts with PAK4 to facilitate the ubiquitination and degradation of aS, thus inhibiting its abnormal aggregation, limiting intercellular propagation, and exerting a neuroprotective effect on dopaminergic neurons. Conversely, NEDD4-1 inhibition disrupts the PAK4-mediated aS clearance pathway (Won et al., 2022). Additionally, NEDD4-1 mediates Lys-63-linked ubiquitination of aS, promoting its targeted trafficking to the endosomal system, enhancing aS degradation, and effectively suppressing its aggregation and intercellular spread (Sugeno et al., 2014). Notably, Ndfip1, an adaptor protein of NEDD4-1, has been shown to exert similar neuroprotective effects against aS-related neurotoxicity (Xing et al., 2016).

### 6.2 Cardiovascular diseases

NEDD4-1 plays a critical role in the onset and progression of cardiovascular diseases, with its dysfunction closely linked to hypertension, atherosclerosis, and myocardial injury (Li et al., 2022; Chen et al., 2024). In injury-induced arterial lesions and atherosclerotic progression, the upregulation of Tudor-SN expression promotes NEDD4-1-mediated polyubiquitination and degradation of PTEN, exacerbating pathological vascular remodeling (Wu et al., 2024). Notably, the PTEN-specific inhibitor BpV (HOpic) effectively reverses the protective effects of Tudor-SN deficiency on vascular smooth muscle cell proliferation and migration, as well as abnormal vascular remodeling induced by carotid artery injury in mice. Hepatocellular Lcn2 enhances HDL metabolism and alleviates atherosclerosis by inhibiting NEDD4-1-mediated ubiquitination of scavenger receptor class B type 1 (SR-BI) (Hu et al., 2023). In myocardial ischemia/reperfusion injury, NEDD4-1 mitigates cardiomyocyte apoptosis by activating the PI3K/Akt signaling pathway (Hu et al., 2017). Furthermore, NEDD4-1 is involved in regulating cardiac ion channels, with LITAF modulating cardiac excitability through NEDD4-1-mediated ubiquitination and degradation of L-type calcium channels (Moshal et al., 2019).

### 6.3 Metabolic diseases and other diseases

In diabetes mellitus and its complications, NEDD4-1 regulates insulin signaling and contributes to the pathogenesis of neuropathy (Chandrasekaran et al., 2019). In obesity-related insulin resistance, Sonic hedgehog (Shh) signaling mediates NEDD4-1-driven degradation of peroxisome proliferator-activated receptor γ (PPARγ), impairing insulin sensitivity (Yao et al., 2019). In diabetic peripheral neuropathy, NAD^+^ precursors activate sirtuin 1 (SIRT1) to facilitate NEDD4-1 deacetylation, promoting axonal regeneration and recovery of nerve function (Chandrasekaran et al., 2024). In skeletal muscle atrophy, NEDD4-1 mediates the ubiquitination of PDZ and LIM domain 7 (PDLIM7) and denervation-induced muscle protein degradation, contributing to muscle fiber cross-sectional area reduction and accelerating the progression of muscle atrophy (D’Cruz et al., 2016; Nagpal et al., 2012). In lymphangiectasia and protein-losing enteropathy, the Nedd4-1 E46Q mutant (NE) exhibits reduced ubiquitin ligase activity toward vacuolar protein sorting 13B (VPS13B), resulting in dysregulated autophagy (Kefalas et al., 2025). In infectious diseases, NEDD4-1 promotes the accumulation of K63-linked polyubiquitin chains (K63Ub) on pneumococcal-containing autophagic vesicles (PcAV), thereby enhancing *Streptococcus* pneumoniae clearance and demonstrating its pivotal role in antibacterial autophagy (Ogawa et al., 2018). In liver disorders, NEDD4-1 protects hepatocytes from acetaminophen-induced injury by targeting voltage-dependent anion channel 1 (VDAC1) for degradation (Zhu et al., 2023). Additionally, NEDD4-1 may play a role in keloid formation through regulation of the PTEN/PI3K/AKT signaling pathway (Sang et al., 2015).

### 6.4 Viruses

NEDD4-1 is exploited by several viruses, including paramyxoviruses, tiger frog virus (TFV), avian reovirus (ARV), HIV, Marburg virus (MARV), murine leukemia virus (MLV), and human T-cell leukemia virus type 1 (HTLV-1), to promote viral egress by recruiting ESCRT or modulating host immune responses (Ray et al., 2019; Chaturbhuj et al., 2018; Mi et al., 2016; Huang et al., 2015; Sette et al., 2010; Urata and Yasuda, 2010; Segura-Morales et al., 2005; Blot et al., 2004). The central role of NEDD4-1 in the pathogenesis of non-neoplastic diseases is summarized in Figure 5.

**FIGURE 5:**
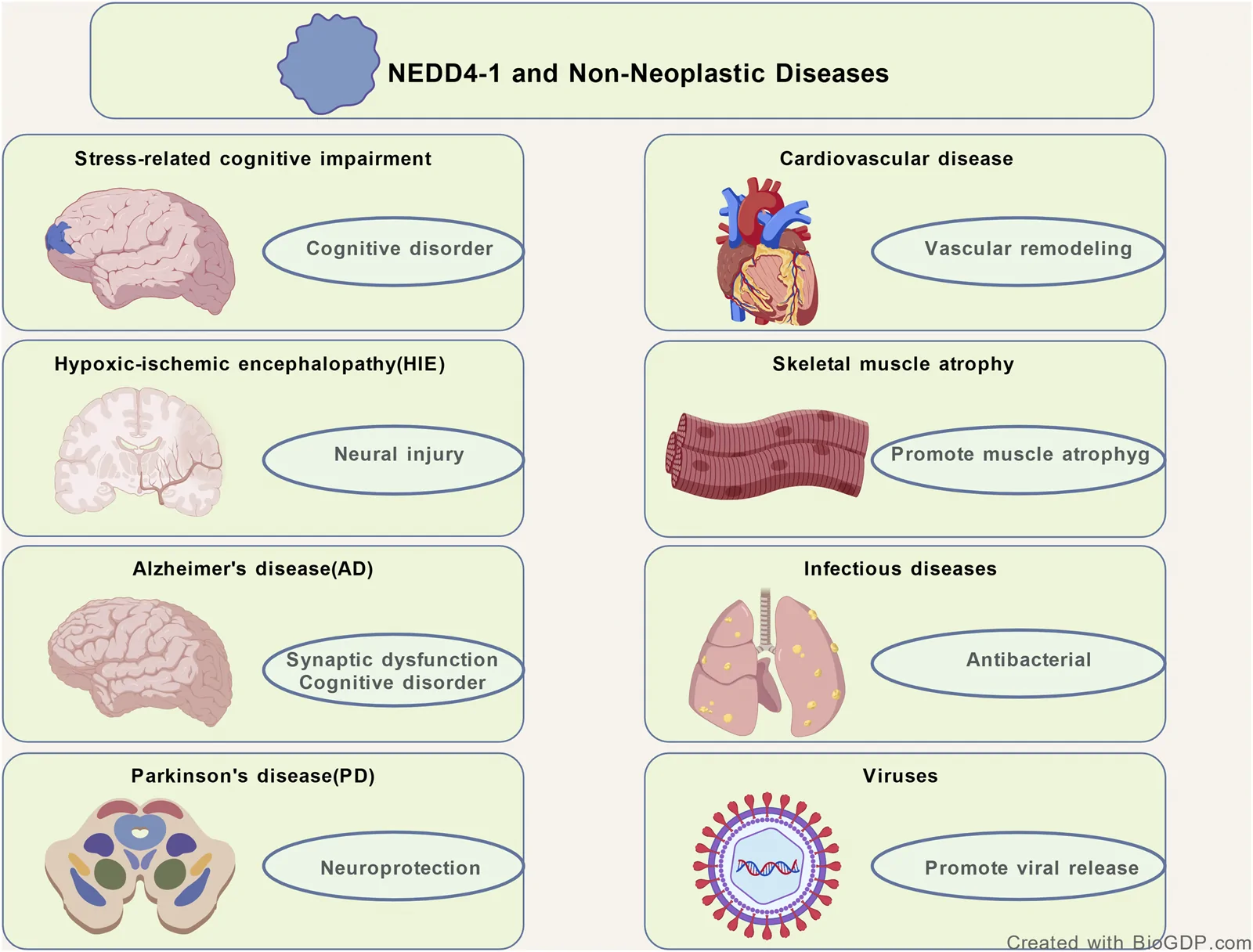
Summary of the roles of NEDD4-1 in non-neoplastic diseases. NEDD4-1 participates in the patho-logical processes of neurological disorders, cardiovascular diseases, skeletal muscle atrophy and infectious diseases, including cognitive dysfunction, neural injury, vascular remodeling, muscle atrophy, antibacterial response and viral release regulation. Created with BioGDP.

## 7 Targeted therapy for NEDD4-1

### 7.1 Inhibitor development

NEDD4-1 inhibitors include natural compounds, small-molecule inhibitors, and peptide inhibitors (Figure 6A) (Bjij et al., 2019a). Among natural products, I3C and its more potent derivative 3,3′-diindolylmethane (DIM) directly bind to the HECT domain of NEDD4-1, inhibiting its ubiquitin ligase activity and demonstrating significant antitumor efficacy in melanoma and other cancer models (Aronchik et al., 2014; Quirit et al., 2017). In glioma models, combination therapy with I3C (a NEDD4-1 inhibitor) and TMZ exhibits synergistic antitumor effects in both *in vitro* and *in vivo* assays, effectively restoring drug sensitivity in TMZ-resistant tumor cells (Chuang et al., 2021). These results highlight I3C and its analogues as promising anticancer agents with clinical potential for targeting NEDD4-1-overexpressing cancers. Another natural compound, lycorine, suppresses bladder cancer cell proliferation and invasiveness by downregulating NEDD4-1 expression (Yang et al., 2022). In recent years, covalent inhibitors have become a key area of focus, specifically targeting cysteine residues within the catalytic domain of NEDD4-1 to alter its catalytic mechanism (Yan et al., 2022; Chen et al., 2024). Rational drug design efforts are prioritizing the development of covalent inhibitors targeting specific cysteine residues in the HECT domain, aiming to switch NEDD4-1-mediated ubiquitination from continuous to stepwise catalysis (Bjij et al., 2019a; Bjij et al., 2019b; Kathman et al., 2015). For example, compound Zinc00937975 effectively inhibits NEDD4-1 activity through covalent binding to its signature cysteine residue, indicating its potential for further drug development (Bjij et al., 2019a). Additionally, blocking peptides and small molecules targeting the CaV3.2-USP5 interaction may provide a therapeutic approach for neuropathic pain by modulating NEDD4-1-mediated degradation of VGCCs (Liu et al., 2025).

**FIGURE 6:**
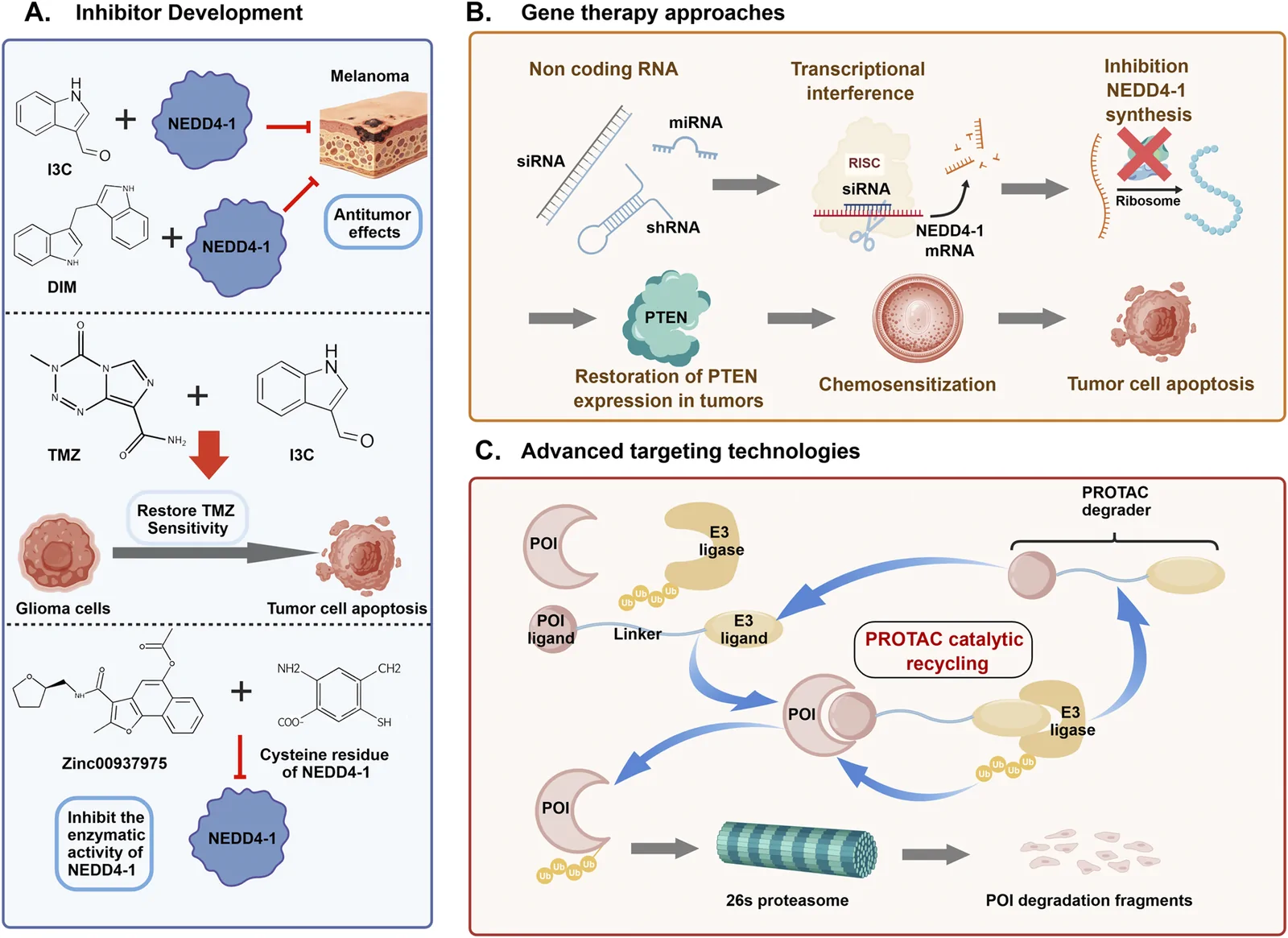
Targeted therapeutic strategies for NEDD4-1. Multiple approaches are currently being developed to modulate NEDD4-1 activity for therapeutic purposes: **(A)** Small-molecule inhibitors: Natural compounds such as indole-3-carbinol (I3C) and its derivative 3,3′-diindolylmethane (DIM) directly bind to the HECT domain, inhibiting ubiquitin transfer. These compounds exhibit antitumor effects in melanoma and glioma models and restore sensitivity to temozolomide in resistant cells. Covalent inhibitors (e.g., Zinc00937975) specifically target the catalytic cysteine within the HECT domain, shifting the ubiquitination mechanism from persistent to distributive. **(B)** Gene therapy approaches and targeting technologies: siRNA/shRNA-mediated NEDD4-1 knockdown restores PTEN expression in tumors, enhancing chemotherapy sensitivity, while NEDD4-1 overexpression promotes α-synuclein degradation in neurodegenerative diseases. Degradation hijacking strategies use bispecific nanobodies to recruit endogenous NEDD4-1/2 onto specific membrane proteins (e.g., KCNQ1, ENaC, CaV2.2), enabling degradation of previously “undruggable” targets. PROTAC technology provides a similar approach for targeting protein degradation. I3C: Indole-3-carbinol; DIM: 3,3′-diindolylmethane; PROTAC: Proteolysis-targeting chimera.

### 7.2 Gene therapy and targeted delivery

Gene therapy strategies are being developed to modulate NEDD4-1 expression and intervene in disease progression (Figure 6B) (Cabezas et al., 2023). In tumors, siRNA- or shRNA-mediated knockdown of NEDD4-1 restores PTEN activity, enhancing chemosensitivity (Henry et al., 2003; Sun H. et al., 2017). In contrast, in neurodegenerative diseases, upregulation of NEDD4-1 accelerates α-synuclein degradation and mitigates its aggregation (Won et al., 2022). Additionally, the advancement of PROTAC technology has spurred the design of therapeutic strategies that recruit E3 ligases to promote the degradation of pathogenic proteins. Innovative “degradation hijacking” approaches, including the use of bivalent nanobodies to redirect endogenous NEDD4-1/2 to specific membrane ion channels (such as multi-subunit KCNQ1, ENaC, and CaV2.2), represent a major breakthrough in targeting proteins previously considered “undruggable” (Morgenstern et al., 2024).

## 8 Conclusions and future perspective

Research on NEDD4-1 has evolved from a focus on linear signaling pathways to the realm of network biology. This molecule is no longer viewed solely as an oncoprotein or tumor suppressor but as a highly integrated cellular signal processor, with its output deeply influenced by both intracellular and extracellular inputs.

Future research should prioritize the following key areas. First, mapping the “interaction landscape” of NEDD4-1: By leveraging high-throughput proteomics, efforts should focus on systematically identifying all interacting proteins, substrates, and regulatory factors within specific disease models and cell types, thereby constructing a comprehensive regulatory network. Second, an in-depth exploration of NEDD4-1 isoform functions: A particular emphasis on the distinct roles of isoforms, such as NEDD4-1(NE), in both physiological and pathological contexts could reveal more precise therapeutic targets. Third, addressing the clinical challenges of NEDD4-1-targeted agents: The poor specificity and off-target effects of current compounds impede their clinical development, highlighting the need for the design of more efficient, specific inhibitors and optimized delivery systems. To address these challenges, integrated approaches are essential: combining single-cell sequencing and spatial proteomics to analyze cell-type-specific expression patterns and functional roles of NEDD4-1 in disease; utilizing gene editing to develop more refined *in vivo* models for investigating regulatory mechanisms; and advancing novel NEDD4-1 inhibitors through high-throughput screening, followed by rigorous preclinical and clinical evaluations to provide innovative therapeutic options for related diseases.

## References

[B1] Ahn Y. Hwang C. Y. Lee S. Kwon K. S. (2008). The tumour suppressor PTEN mediates a negative regulation of the E3 ubiquitin-protein ligase Nedd4. Biochem. J. 412 (2), 331–338. 10.1042/BJ20071403 18307411

[B2] Akkaya B. G. Zolnerciks J. K. Ritchie T. K. Bauer B. Hartz A. M. S. Sullivan J. A. (2015). The multidrug resistance pump ABCB1 is a substrate for the ubiquitin ligase NEDD4-1. Mol. Membr. Biol. 32 (2), 39–45. 10.3109/09687688.2015.1023378 26006083 PMC4844833

[B3] Alrosan A. Z. Heilat G. B. Alrosan K. Shannag A. Alshalout E. M. (2025). NEDD4 signaling: a new frontier in the diagnosis and treatment of breast and ovarian cancers. Med. Oncol. 42 (6), 200. 10.1007/s12032-025-02751-z 40327180

[B4] Altas B. Rhee H. J. Ju A. Solís H. C. Karaca S. Winchenbach J. (2024). Nedd4-2-dependent regulation of astrocytic Kir4.1 and Connexin43 controls neuronal network activity. J. Cell Biol. 223 (1), e201902050. 10.1083/jcb.201902050 38032389 PMC10689203

[B5] Amodio N. Scrima M. Palaia L. Salman A. N. Quintiero A. Franco R. (2010). Oncogenic role of the E3 ubiquitin ligase NEDD4-1, a PTEN negative regulator, in non-small-cell lung carcinomas. Am. J. Pathol. 177 (5), 2622–2634. 10.2353/ajpath.2010.091075 20889565 PMC2966817

[B6] Aronchik I. Kundu A. Quirit J. G. Firestone G. L. (2014). The antiproliferative response of indole-3-carbinol in human melanoma cells is triggered by an interaction with NEDD4-1 and disruption of wild-type PTEN degradation. Mol. Cancer Res. 12 (11), 1621–1634. 10.1158/1541-7786.MCR-14-0018 25009292 PMC4233179

[B7] Bachofner M. Speicher T. Bogorad R. L. Muzumdar S. Derrer C. P. Hürlimann F. (2017). Large-scale quantitative proteomics identifies the ubiquitin ligase Nedd4-1 as an essential regulator of liver regeneration. Dev. Cell 42 (6), 616–625. 10.1016/j.devcel.2017.07.025 28890072

[B8] Behrends C. Sowa M. E. Gygi S. P. Harper J. W. (2010). Network organization of the human autophagy system. Nature 466 (7302), 68–76. 10.1038/nature09204 20562859 PMC2901998

[B9] Bellet M. M. Piobbico D. Bartoli D. Castelli M. Pieroni S. Brunacci C. (2014). NEDD4 controls the expression of GUCD1, a protein upregulated in proliferating liver cells. Cell Cycle 13 (12), 1902–1911. 10.4161/cc.28760 24743017 PMC4111753

[B10] Bjij I. Khan S. Betz R. Cherqaoui D. Soliman M. E. S. (2018). Exploring the structural mechanism of covalently bound E3 ubiquitin ligase: catalytic or allosteric inhibition? Protein J. 37 (6), 500–509. 10.1007/s10930-018-9795-5 30232697

[B11] Bjij I. Ramharack P. Khan S. Cherqaoui D. Soliman M. E. S. (2019a). Tracing potential covalent inhibitors of an E3 ubiquitin ligase through target-focused modelling. Molecules 24 (17), 3125. 10.3390/molecules24173125 31466292 PMC6749425

[B12] Bjij I. Khan S. Ramharak P. Cherqaoui D. Soliman M. E. S. (2019b). Distinguishing the optimal binding mechanism of an E3 ubiquitin ligase: covalent versus noncovalent inhibition. J. Cell Biochem. 120 (8), 12859–12869. 10.1002/jcb.28556 30854719

[B13] Blot V. Perugi F. Gay B. Prévost M. C. Briant L. Tangy F. (2004). Nedd4.1-mediated ubiquitination and subsequent recruitment of Tsg101 ensure HTLV-1 gag trafficking towards the multivesicular body pathway prior to virus budding. J. Cell Sci. 117 (11), 2357–2367. 10.1242/jcs.01095 15126635

[B14] Bobby R. Medini K. Neudecker P. Lee T. V. Brimble M. A. McDonald F. J. (2013). Structure and dynamics of human Nedd4-1 WW3 in complex with the αENaC PY motif. Biochim. Biophys. Acta 1834 (8), 1632–1641. 10.1016/j.bbapap.2013.04.031 23665454

[B15] Booth L. Roberts J. L. Poklepovic A. Dent P. (2018). NEDD4 over-expression regulates the afatinib resistant phenotype of NSCLC cells. Oncol. Signal. 1 (1), 19–30. 10.1016/j.onsig.2017.07.001 30740589 PMC6366836

[B16] Cabezas F. Cabello-Verrugio C. González N. Salas J. Ramírez M. J. de la Vega E. (2023). NEDD4-1 deficiency impairs satellite cell function during skeletal muscle regeneration. Biol. Res. 56 (1), 21. 10.1186/s40659-023-00432-7 37147738 PMC10161651

[B17] Chai A. B. Callaghan R. Gelissen I. C. (2022). The ubiquitin E3 ligase Nedd4 regulates the expression and Amyloid-β peptide export activity of P-Glycoprotein. Int. J. Mol. Sci. 23 (3), 1019. 10.3390/ijms23031019 35162941 PMC8834788

[B18] Chandrasekaran K. Salimian M. Konduru S. R. Choi J. Kumar P. Long A. (2019). Overexpression of sirtuin 1 protein in neurons prevents and reverses experimental diabetic neuropathy. Brain 142 (12), 3737–3752. 10.1093/brain/awz324 31754701 PMC6885680

[B19] Chandrasekaran K. Najimi N. Sagi A. R. Yarlagadda S. Salimian M. Arvas M. I. (2024). NAD(+) precursors reverse experimental diabetic neuropathy in mice. Int. J. Mol. Sci. 25 (2), 1102. 10.3390/ijms25021102 38256175 PMC10816262

[B20] Chaturbhuj D. Patil A. Gangakhedkar R. (2018). PYRE insertion within HIV-1 subtype C p6-Gag functions as an ALIX-dependent late domain. Sci. Rep. 8 (1), 8917. 10.1038/s41598-018-27162-1 29891975 PMC5995805

[B21] Chen Y. van de Vijver M. J. Hibshoosh H. Parsons R. Saal L. H. (2016). PTEN and NEDD4 in human breast carcinoma. Pathol. Oncol. Res. 22 (1), 41–47. 10.1007/s12253-015-9971-2 26276352 PMC4681749

[B22] Chen X. Ma J. Wang Z.-W. Wang Z. (2024). The E3 ubiquitin ligases regulate inflammation in cardiovascular diseases. Semin. Cell Dev. Biol. 154 (Pt C), 167–174. 10.1016/j.semcdb.2023.02.008 36872193

[B23] Cheng F. Cheng Y. Zhao X. An L. Yang L. Li Z. (2021). NEDD4 E3 ubiquitin protein ligase serves an important role in cutaneous melanoma occurrence and development. Exp. Ther. Med. 22 (6), 1382. 10.3892/etm.2021.10818 34650630 PMC8506948

[B24] Choi K. M. Kim S. J. Ji M. J. Kim E. Kim J. S. Park H. (2024). Activity-based protein profiling and global proteome analysis reveal MASTL as a potential therapeutic target in gastric cancer. Cell Commun. Signal 22 (1), 397. 10.1186/s12964-024-01783-8 39138495 PMC11323684

[B25] Chuang H. Y. Hsu L. Y. Pan C. M. Pikatan N. W. Yadav V. K. Fong I. H. (2021). The E3 ubiquitin ligase NEDD4-1 mediates temozolomide-resistant glioblastoma through PTEN attenuation and redox imbalance in Nrf2-HO-1 axis. Int. J. Mol. Sci. 22 (19), 10247. 10.3390/ijms221910247 34638586 PMC8549703

[B26] D'Cruz R. Plant P. J. Pablo L. A. Lin S. Chackowicz J. Correa J. (2016). PDLIM7 is a novel target of the ubiquitin ligase Nedd4-1 in skeletal muscle. Biochem. J. 473 (3), 267–276. 10.1042/BJ20150222 26556890

[B27] Dai B. Pieper R. O. Li D. Wei P. Liu M. Woo S. Y. (2010). FoxM1B regulates NEDD4-1 expression, leading to cellular transformation and full malignant phenotype in immortalized human astrocytes. Cancer Res. 70 (7), 2951–2961. 10.1158/0008-5472.CAN-09-3909 20332230 PMC2848915

[B28] Dai C. Wu B. Chen Y. Li X. Bai Y. Du Y. (2021). Aagab acts as a novel regulator of NEDD4-1-mediated pten nuclear translocation to promote neurological recovery following hypoxic-ischemic brain damage. Cell Death Differ. 28 (8), 2367–2384. 10.1038/s41418-021-00757-4 33712741 PMC8328997

[B29] Drinjakovic J. Jung H. Campbell D. S. Strochlic L. Dwivedy A. Holt C. E. (2010). E3 ligase Nedd4 promotes axon branching by downregulating PTEN. Neuron 65 (3), 341–357. 10.1016/j.neuron.2010.01.017 20159448 PMC2862300

[B30] Fang S. Peng L. Zhang M. Hou R. Deng X. Li X. (2024). MiR-2110 induced by chemically synthesized cinobufagin functions as a tumor-metastatic suppressor via targeting FGFR1 to reduce PTEN ubiquitination degradation in nasopharyngeal carcinoma. Environ. Toxicol. 39 (6), 3548–3562. 10.1002/tox.24197 38477013

[B31] Feng S. Yang G. Yang H. Liang Z. Zhang R. Fan Y. (2017). NEDD4 is involved in acquisition of epithelial-mesenchymal transition in cisplatin-resistant nasopharyngeal carcinoma cells. Cell Cycle (Georgetown, Tex.) 16 (9), 869–878. 10.1080/15384101.2017.1308617 28379054 PMC5444352

[B32] Fouladkou F. Lu C. Jiang C. Zhou L. She Y. Walls J. R. (2010). The ubiquitin ligase Nedd4-1 is required for heart development and is a suppressor of thrombospondin-1. J. Biol. Chem. 285 (9), 6770–6780. 10.1074/jbc.M109.082347 20026598 PMC2825471

[B33] Gao R. Bian Y. Wang Y. Zhang Y. Zhu Q. Nie J. (2025). NEDD4 is a biomarker of a poor prognosis that contributes to the progression and chemoresistance in small cell lung cancer. Cell cycle (Georgetown, Tex.) 24 (9-12), 204–219. 10.1080/15384101.2025.2539657 40767383 PMC12427450

[B34] Garcia-Cao I. Song M. S. Hobbs R. M. Laurent G. Giorgi C. de Boer V. C. J. (2012). Systemic elevation of PTEN induces a tumor-suppressive metabolic state. Cell 149 (1), 49–62. 10.1016/j.cell.2012.02.030 22401813 PMC3319228

[B35] Gorla M. Chaudhari K. Hale M. Potter C. Bashaw G. J. (2022). A Nedd4 E3 ubiquitin ligase pathway inhibits Robo1 repulsion and promotes commissural axon guidance across the midline. J. Neurosci. 42 (40), 7547–7561. 10.1523/JNEUROSCI.2491-21.2022 36002265 PMC9546450

[B36] Guntupalli S. Jang S. E. Zhu T. Huganir R. L. Widagdo J. Anggono V. (2017). GluA1 subunit ubiquitination mediates amyloid-β-induced loss of surface α-amino-3-hydroxy-5-methyl-4-isoxazolepropionic acid (AMPA) receptors. J. Biol. Chem. 292 (20), 8186–8194. 10.1074/jbc.M116.774554 28377502 PMC5437227

[B37] Han J. Shin Y. Kim E. Park H. M. Kim J. Y. (2025). Proteomic characterization of NEDD4 unveils its potential novel downstream effectors in gastric cancer. J. Proteome Res. 24 (2), 891–902. 10.1021/acs.jproteome.4c01109 39874481

[B38] Henry P. C. Kanelis V. O'Brien M. C. Kim B. Gautschi I. Forman-Kay J. (2003). Affinity and specificity of interactions between Nedd4 isoforms and the epithelial na+ channel. J. Biol. Chem. 278 (22), 20019–20028. 10.1074/jbc.M211153200 12654927

[B39] Hirota Y. Hayashi M. Miyauchi Y. Ishii Y. Tanaka Y. Fujimoto K. (2021). LAPTM4α is targeted from the golgi to late endosomes/lysosomes in a manner dependent on the E3 ubiquitin ligase Nedd4-1 and ESCRT proteins. Biochem. Biophys. Res. Commun. 556, 9–15. 10.1016/j.bbrc.2021.03.151 33836347

[B40] Hong S. Moon J. Kim J. Shin J. S. Jung K. A. Lee W. K. (2014). p34 is a novel regulator of the oncogenic behavior of NEDD4-1 and PTEN. Cell Death Differ. 21 (1), 146–160. 10.1038/cdd.2013.141 24141722 PMC3857621

[B41] Hsia H. E. Kawabe H. (2015). Translational regulation of the expression of the ubiquitin E3 ligase Nedd4-1 by mTORC1-dependent signaling. Commun. Integr. Biol. 8 (4), e1017161. 10.1080/19420889.2015.1017161 26479408 PMC4594434

[B42] Hsia H. E. Kumar R. Luca R. Takeda M. Courchet J. Nakashima J. (2014). Ubiquitin E3 ligase Nedd4-1 acts as a downstream target of PI3K/PTEN-mTORC1 signaling to promote neurite growth. Proc. Natl. Acad. Sci. U. S. A. 111 (36), 13205–13210. 10.1073/pnas.1400737111 25157163 PMC4246980

[B43] Hsu W. L. Ma Y. L. Chen Y. C. Liu Y. C. Cheng K. M. Lee E. H. Y. (2023). Identification of Ndfip1 as a novel negative regulator for spatial memory formation associated with increased ubiquitination of beclin 1 and PTEN. PLoS One 18 (4), e0283908. 10.1371/journal.pone.0283908 PMC10079018 37023120

[B44] Hu W. Zhang P. Gu J. Yu Q. Zhang D. (2017). NEDD4-1 protects against ischaemia/reperfusion-induced cardiomyocyte apoptosis via the PI3K/Akt pathway. Apoptosis 22 (3), 437–448. 10.1007/s10495-016-1326-2 27837380

[B45] Hu S. Zhu Y. Zhao X. Li R. Shao G. Gong D. (2023). Hepatocytic lipocalin-2 controls HDL metabolism and atherosclerosis via Nedd4-1-SR-BI axis in mice. Dev. Cell 58 (21), 2326–2337. 10.1016/j.devcel.2023.09.007 37863040

[B46] Huang W. R. Chiu H. C. Liao T. L. Chuang K. P. Shih W. L. Liu H. J. (2015). Avian reovirus protein p17 functions as a nucleoporin tpr suppressor leading to activation of p53, p21 and PTEN and inactivation of PI3K/AKT/mTOR and ERK signaling pathways. PLoS One 10 (8), e0133699. 10.1371/journal.pone.0133699 26244501 PMC4526660

[B47] Huang Z. Zhu J. Yang X. Biskup E. (2017). NEDD4 promotes cell growth and migration via PTEN/PI3K/AKT signaling in hepatocellular carcinoma. Oncol. Let. 14 (3), 2649–2656. 10.3892/ol.2017.6532 28928809 PMC5588169

[B48] Huang X. Chen J. Cao W. Yang L. Chen Q. He J. (2019). The many substrates and functions of NEDD4-1. Cell Death Dis. 10 (12), 904. 10.1038/s41419-019-2142-8 31787758 PMC6885513

[B49] Huang X. Gu H. Zhang E. Chen Q. Cao W. Yan H. (2020). The NEDD4-1 E3 ubiquitin ligase: a potential molecular target for bortezomib sensitivity in multiple myeloma. Int. J. Cancer 146 (7), 1963–1978. 10.1002/ijc.32615 31390487 PMC7027789

[B50] Jiang K. Bai B. Ta Y. Zhang T. Xiao Z. Wang P. G. (2016). O-GlcNAc regulates NEDD4-1 stability via caspase-mediated pathway. Biochem. Biophys. Res. Commun. 471 (4), 539–544. 10.1016/j.bbrc.2016.02.037 26876577

[B51] Jiang H. Thomas S. N. Chen Z. Chiang C. Y. Cole P. A. (2019). Comparative analysis of the catalytic regulation of NEDD4-1 and WWP2 ubiquitin ligases. J. Biol. Chem. 294 (46), 17421–17436. 10.1074/jbc.RA119.009211 31578285 PMC6873185

[B52] Jung S. Li C. Jeong D. Lee S. Ohk J. Park M. (2013). Oncogenic function of p34SEI-1 via NEDD4-1-mediated PTEN ubiquitination/degradation and activation of the PI3K/AKT pathway. Int. J. Oncol. 43 (5), 1587–1595. 10.3892/ijo.2013.2064 23970032

[B53] Kathman S. G. Span I. Smith A. T. Xu Z. Zhan J. Rosenzweig A. C. (2015). A small molecule that switches a ubiquitin ligase from a processive to a distributive enzymatic mechanism. J. Am. Chem. Soc. 137 (39), 12442–12445. 10.1021/jacs.5b06839 26371805 PMC4669213

[B54] Kawabe H. Brose N. (2010). The ubiquitin E3 ligase Nedd4-1 controls neurite development. Cell Cycle (Georgetown, Tex.) 9 (13), 2477–2478. 10.4161/cc.9.13.12236 20543564

[B55] Kawabe H. Neeb A. Dimova K. Young S. M. Takeda M. Katsurabayashi S. (2010). Regulation of Rap2A by the ubiquitin ligase Nedd4-1 controls neurite development. Neuron 65 (3), 358–372. 10.1016/j.neuron.2010.01.007 20159449 PMC2825371

[B56] Kefalas G. Rotin D. (2023). Primate-specific isoform of Nedd4-1 regulates substrate binding via ser/thr phosphorylation and 14-3-3 binding. Sci. Rep. 13 (1), 17903. 10.1038/s41598-023-44761-9 37863970 PMC10589272

[B57] Kefalas G. Priya A. Astori A. Persaud A. Jing L. Sydor A. M. (2025). The primate-specific Nedd4-1(NE) localizes to late endosomes in response to amino acids to suppress autophagy. Nat. Commun. 16 (1), 2682. 10.1038/s41467-025-57944-x 40102426 PMC11920435

[B58] Kim S. S. Yoo N. J. Jeong E. G. Kim M. S. Lee S. H. (2008). Expression of NEDD4-1, a PTEN regulator, in gastric and colorectal carcinomas. APMIS 116 (9), 779–784. 10.1111/j.1600-0463.2008.00999.x 19024597

[B59] Kwak Y. D. Ma T. Diao S. Zhang X. Chen Y. Hsu J. (2010). NO signaling and S-nitrosylation regulate PTEN inhibition in neurodegeneration. Mol. Neurodegener. 5, 49. 10.1186/1750-1326-5-49 21067594 PMC2992530

[B60] Lackovic J. Howitt J. Callaway J. K. Silke J. Bartlett P. Tan S. S. (2012). Differential regulation of Nedd4 ubiquitin ligases and their adaptor protein Ndfip1 in a rat model of ischemic stroke. Exp. Neurol. 235 (1), 326–335. 10.1016/j.expneurol.2012.02.014 22417925

[B61] Li Z. Li J. Bi P. Lu Y. Burcham G. Elzey B. D. (2014). Plk1 phosphorylation of PTEN causes a tumor-promoting metabolic state. Mol. Cell. Biol. 34 (19), 3642–3661. 10.1128/MCB.00814-14 25047839 PMC4187734

[B62] Li Y. Zhang L. Zhou J. Luo S. Huang R. Zhao C. (2015). Nedd4 E3 ubiquitin ligase promotes cell proliferation and autophagy. Cell Prolifer. 48 (3), 338–347. 10.1111/cpr.12184 25809873 PMC6495840

[B63] Li M. Sun G. Wang P. Wang W. Cao K. Song C. (2022). Research progress of Nedd4L in cardiovascular diseases. Cell Death Discov. 8 (1), 206. 10.1038/s41420-022-01017-1 35429991 PMC9013375

[B64] Lin Q. Dai Q. Meng H. Sun A. Wei J. Peng K. (2017). The HECT E3 ubiquitin ligase NEDD4 interacts with and ubiquitylates SQSTM1 for inclusion body autophagy. J. Cell Sci. 130 (22), 3839–3850. 10.1242/jcs.207068 29021346

[B65] Liu L. Zhou Y. Liao P. Soong T. W. Hu Z. (2025). Degradation of voltage-gated calcium channels: mechanisms and applications in neurological and cardiovascular diseases. Cell Commun. Signal 23 (1), 337. 10.1186/s12964-025-02347-0 40660291 PMC12261568

[B66] Lu C. Thoeni C. Connor A. Kawabe H. Gallinger S. Rotin D. (2016). Intestinal knockout of Nedd4 enhances growth of apc(min) tumors. Oncogene 35 (45), 5839–5849. 10.1038/onc.2016.125 27086928

[B67] Mi S. Qin X. W. Lin Y. F. He J. Chen N. N. Liu C. (2016). Budding of tiger frog virus (an iridovirus) from HepG2 cells via three ways recruits the ESCRT pathway. Sci. Rep. 6, 26581. 10.1038/srep26581 27225426 PMC4880917

[B68] Morgenstern T. J. Darko-Boateng A. Afriyie E. Shanmugam S. K. Zhou X. Choudhury P. (2024). Ion Channel Inhibition by Targeted Recruitment of NEDD4-2 with Divalent Nanobodies. bioRxiv. 10.1101/2024.05.28.596281 PMC12796476 41353348

[B69] Moshal K. S. Roder K. Kabakov A. Y. Werdich A. A. Chiang D. Y. E. Turan N. N. (2019). LITAF (Lipopolysaccharide-Induced tumor necrosis factor) regulates cardiac L-Type calcium channels by modulating NEDD (neural precursor cell expressed developmentally downregulated protein) 4-1 ubiquitin ligase. Circ. Genom Precis. Med. 12 (9), 407–420. 10.1161/CIRCGEN.119.002641 31462068 PMC6750970

[B70] Nagpal P. Plant P. J. Correa J. Bain A. Takeda M. Kawabe H. (2012). The ubiquitin ligase Nedd4-1 participates in denervation-induced skeletal muscle atrophy in mice. PLoS One 7 (10), e46427. 10.1371/journal.pone.0046427 23110050 PMC3482220

[B71] Ogawa M. Matsuda R. Takada N. Tomokiyo M. Yamamoto S. Shizukuishi S. (2018). Molecular mechanisms of streptococcus pneumoniae-targeted autophagy *via* pneumolysin, Golgi-resident Rab41, and Nedd4-1-mediated K63-linked ubiquitination. Cell Microbiol. 20 (8), e12846. 10.1111/cmi.12846 29582580

[B72] Panwalkar V. Schulte M. Lecher J. Stoldt M. Willbold D. Dingley A. J. (2016a). Data describing the solution structure of the WW3* domain from human Nedd4-1. Data Brief. 8, 605–612. 10.1016/j.dib.2016.06.024 27419198 PMC4936499

[B73] Panwalkar V. Neudecker P. Schmitz M. Lecher J. Schulte M. Medini K. (2016b). The Nedd4-1 WW domain recognizes the PY motif peptide through coupled folding and binding equilibria. Biochemistry 55 (4), 659–674. 10.1021/acs.biochem.5b01028 26685112

[B74] Pei G. Buijze H. Liu H. Moura-Alves P. Goosmann C. Brinkmann V. (2017). The E3 ubiquitin ligase NEDD4 enhances killing of membrane-perturbing intracellular bacteria by promoting autophagy. Autophagy 13 (12), 2041–2055. 10.1080/15548627.2017.1376160 29251248 PMC5788543

[B75] Persaud A. Alberts P. Hayes M. Guettler S. Clarke I. Sicheri F. (2011). Nedd4-1 binds and ubiquitylates activated FGFR1 to control its endocytosis and function. EMBO J. 30 (16), 3259–3273. 10.1038/emboj.2011.234 21765395 PMC3160656

[B76] Persaud A. Alberts P. Mari S. Tong J. Murchie R. Maspero E. (2014). Tyrosine phosphorylation of NEDD4 activates its ubiquitin ligase activity. Sci. Signal 7 (346), ra95. 10.1126/scisignal.2005290 25292214

[B77] Putz U. Howitt J. Doan A. Goh C. P. Low L. H. Silke J. (2012). The tumor suppressor PTEN is exported in exosomes and has phosphatase activity in recipient cells. Sci. Signal 5 (243), ra70. 10.1126/scisignal.2003084 23012657

[B78] Quirit J. G. Lavrenov S. N. Poindexter K. Xu J. Kyauk C. Durkin K. A. (2017). Indole-3-carbinol (I3C) analogues are potent small molecule inhibitors of NEDD4-1 ubiquitin ligase activity that disrupt proliferation of human melanoma cells. Biochem. Pharmacol. 127, 13–27. 10.1016/j.bcp.2016.12.007 27979631

[B79] Ray G. Schmitt P. T. Schmitt A. P. (2019). Angiomotin-like 1 links paramyxovirus M proteins to NEDD4 family ubiquitin ligases. Viruses 11 (2), 128. 10.3390/v11020128 30708959 PMC6410177

[B80] Rodrigues E. M. Scudder S. L. Goo M. S. Patrick G. N. (2016). Aβ-Induced synaptic alterations require the E3 ubiquitin ligase Nedd4-1. J. Neurosci. 36 (5), 1590–1595. 10.1523/JNEUROSCI.2964-15.2016 26843640 PMC4737771

[B81] Rotin D. Prag G. (2024). Physiological functions of the ubiquitin ligases Nedd4-1 and Nedd4-2. Physiol. (Bethesda) 39 (1), 18–29. 10.1152/physiol.00023.2023 37962894

[B82] Rougier J. S. Albesa M. Abriel H. Viard P. (2011). Neuronal precursor cell-expressed developmentally down-regulated 4-1 (NEDD4-1) controls the sorting of newly synthesized Ca(V)1.2 calcium channels. J. Biol. Chem. 286 (11), 8829–8838. 10.1074/jbc.M110.166520 21220429 PMC3059038

[B83] Sang P. F. Wang H. Wang M. Hu C. Zhang J. S. Li X. J. (2015). NEDD4-1 and PTEN expression in keloid scarring. Genet. Mole.r Res. GMR 14 (4), 13467–13475. 10.4238/2015.October.28.7 26535660

[B84] Schulte M. Panwalkar V. Freischem S. Willbold D. Dingley A. J. (2018). Proline restricts loop I conformation of the high affinity WW domain from human Nedd4-1 to a ligand binding-competent type I β-Turn. J. Phys. Chem. B 122 (15), 4219–4230. 10.1021/acs.jpcb.7b11637 29595969

[B85] Schwarz L. A. Hall B. J. Patrick G. N. (2010). Activity-dependent ubiquitination of GluA1 mediates a distinct AMPA receptor endocytosis and sorting pathway. J. Neurosci. 30 (49), 16718–16729. 10.1523/JNEUROSCI.3686-10.2010 21148011 PMC3079366

[B86] Scudder S. L. Goo M. S. Cartier A. E. Molteni A. Schwarz L. A. Wright R. (2014). Synaptic strength is bidirectionally controlled by opposing activity-dependent regulation of Nedd4-1 and USP8. J. Neurosci. 34 (50), 16637–16649. 10.1523/JNEUROSCI.2452-14.2014 25505317 PMC4261092

[B87] Segura-Morales C. Pescia C. Chatellard-Causse C. Sadoul R. Bertrand E. Basyuk E. (2005). Tsg101 and alix interact with murine leukemia virus gag and cooperate with Nedd4 ubiquitin ligases during budding. J. Biol. Chem. 280 (29), 27004–27012. 10.1074/jbc.M413735200 15908698

[B88] Sette P. Jadwin J. A. Dussupt V. Bello N. F. Bouamr F. (2010). The ESCRT-associated protein alix recruits the ubiquitin ligase Nedd4-1 to facilitate HIV-1 release through the LYPXnL L domain motif. J. Virol. 84 (16), 8181–8192. 10.1128/JVI.00634-10 20519395 PMC2916511

[B89] Shao C. Li Z. Ahmad N. Liu X. (2017). Regulation of PTEN degradation and NEDD4-1 E3 ligase activity by numb. Cell Cycle (Georgetown, Tex.) 16 (10), 957–967. 10.1080/15384101.2017.1310351 28437168 PMC5462079

[B90] Shao G. Wang R. Sun A. Wei J. Peng K. Dai Q. (2018). The E3 ubiquitin ligase NEDD4 mediates cell migration signaling of EGFR in lung cancer cells. Mol. Cancer 17 (1), 24. 10.1186/s12943-018-0784-2 29455656 PMC5817799

[B91] Shrestha P. Yun J. Ko Y. Yeon K. J. Kim D. Lee H. (2017). NMR uncovers direct interaction between human NEDD4-1 and p34(SEI-1). Biochem. Biophys. Res. Commun. 490 (3), 984–990. 10.1016/j.bbrc.2017.06.151 28666866

[B92] Song Y. H. Zhang C. Q. Chen F. F. Lin X. Y. (2018). Upregulation of neural precursor cell expressed developmentally downregulated 4-1 is associated with poor prognosis and chemoresistance in lung adenocarcinoma. Chin. Med. J. Engl. 131 (1), 16–24. 10.4103/0366-6999.221262 29271375 PMC5754953

[B93] Sugeno N. Hasegawa T. Tanaka N. Fukuda M. Wakabayashi K. Oshima R. (2014). Lys-63-linked ubiquitination by E3 ubiquitin ligase Nedd4-1 facilitates endosomal sequestration of internalized α-synuclein. J. Biol. Chem. 289 (26), 18137–18151. 10.1074/jbc.M113.529461 24831002 PMC4140275

[B94] Sun A. Yu G. Dou X. Yan X. Yang W. Lin Q. (2014). Nedd4-1 is an exceptional prognostic biomarker for gastric cardia adenocarcinoma and functionally associated with metastasis. Mol. Cancer 13, 248. 10.1186/1476-4598-13-248 25395181 PMC4239324

[B95] Sun A. Wei J. Childress C. Shaw J. H. Peng K. Shao G. (2017). The E3 ubiquitin ligase NEDD4 is an LC3-interactive protein and regulates autophagy. Autophagy 13 (3), 522–537. 10.1080/15548627.2016.1268301 28085563 PMC5361608

[B96] Sun H. Ma H. Wang J. Xia L. Zhu G. Wang Z. (2017). Phosphatase and tensin homolog deleted on chromosome 10 degradation induced by NEDD4 promotes acquired erlotinib resistance in non-small-cell lung cancer. Tumour Biol. 39 (7), 1393380025. 10.1177/1010428317709639 28714370

[B97] Teafatiller T. Perez O. Kitazawa M. Agrawal A. Subramanian V. S. (2023). Nedd4-1 regulates human sodium-dependent vitamin C transporter-2 functional expression in neuronal and epithelial cells. J. Nutr. Biochem. 120, 109413. 10.1016/j.jnutbio.2023.109413 37423323

[B98] Urata S. Yasuda J. (2010). Regulation of marburg virus (MARV) budding by Nedd4.1: a different WW domain of Nedd4.1 is critical for binding to MARV and ebola virus VP40. J. Gen. Virol. 91 (Pt 1), 228–234. 10.1099/vir.0.015495-0 19812267

[B99] van de Vijver M. J. He Y. D. Van'T Veer L. J. Dai H. Hart A. A. M. Voskuil D. W. (2002). A gene-expression signature as a predictor of survival in breast cancer. New England J. Med. 347 (25), 1999–2009. 10.1056/NEJMoa021967 12490681

[B100] vanʼt Veer L. J. Dai H. van de Vijver M. J. He Y. D. Hart A. A. M. Mao M. (2002). Gene expression profiling predicts clinical outcome of breast cancer. Nature 415 (6871), 530–536. 10.1038/415530a 11823860

[B101] Wang X. Trotman L. C. Koppie T. Alimonti A. Chen Z. Gao Z. (2007). NEDD4-1 is a proto-oncogenic ubiquitin ligase for PTEN. Cell 128 (1), 129–139. 10.1016/j.cell.2006.11.039 17218260 PMC1828909

[B102] Wang J. Peng Q. Lin Q. Childress C. Carey D. Yang W. (2010). Calcium activates Nedd4 E3 ubiquitin ligases by releasing the C2 domain-mediated auto-inhibition. J. Biol. Chem. 285 (16), 12279–12288. 10.1074/jbc.M109.086405 20172859 PMC2852967

[B103] Wang L. Zhu B. Wang S. Wu Y. Zhan W. Xie S. (2017). Regulation of glioma migration and invasion via modification of Rap2a activity by the ubiquitin ligase Nedd4-1. Oncol. Rep. 37 (5), 2565–2574. 10.3892/or.2017.5572 28405688 PMC5428538

[B104] Wiszniak S. Alankarage D. Lohraseb I. Marchant C. Secker G. Domingo D. (2026). Neural crest cell-derived DKK1 and NEDD4 modulate wnt signalling in the?second heart field to orchestrate outflow tract development. Nat. Commun. 17, 10–1038. 10.1038/s41467-026-68459-4 41571667 PMC12913648

[B105] Won S. Y. Park J. J. You S. T. Hyeun J. A. Kim H. K. Jin B. K. (2022). p21-activated kinase 4 controls the aggregation of α-synuclein by reducing the monomeric and aggregated forms of α-synuclein: involvement of the E3 ubiquitin ligase NEDD4-1. Cell Death Dis. 13 (6), 575. 10.1038/s41419-022-05030-1 35773260 PMC9247077

[B106] Wu Y. Chen Z. Zheng Z. Li X. Shu J. Mao R. (2024). Tudor-SN exacerbates pathological vascular remodeling by promoting the polyubiquitination of PTEN via NEDD4-1. J. Biomed. Sci. 31 (1), 88. 10.1186/s12929-024-01076-9 39237902 PMC11378411

[B107] Xie W. Jin S. Cui J. (2020a). The NEDD4-USP13 axis facilitates autophagy via deubiquitinating PIK3C3. Autophagy 16 (6), 1150–1151. 10.1080/15548627.2020.1743071 32174250 PMC7469492

[B108] Xie W. Jin S. Wu Y. Xian H. Tian S. Liu D. A. (2020b). Auto-ubiquitination of NEDD4-1 recruits USP13 to facilitate autophagy through deubiquitinating VPS34. Cell Rep. 30 (8), 2807–2819. 10.1016/j.celrep.2020.01.088 32101753

[B109] Xin L. Zhou L. Liu C. Zeng F. Yuan Y. W. Zhou Q. (2021). Transfer of LncRNA CRNDE in TAM-derived exosomes is linked with cisplatin resistance in gastric cancer. EMBO Reports 22 (12), e52124. 10.15252/embr.202052124 34647680 PMC8647143

[B110] Xing L. F. Guo H. P. Wang D. T. Sun L. H. Pan S. Y. (2016). Protective effects and mechanisms of Ndfipl on SH-SY5Y cell apoptosis in an *in vitro* Parkinson's disease model. Genet. Mol. Res. 15 (2), gmr15026963. 10.4238/gmr.15026963 27173227

[B111] Yan S. Ripamonti R. Kawabe H. Ben-Yehuda Greenwald M. Werner S. (2022). NEDD4-1 is a key regulator of epidermal homeostasis and wound repair. J. Invest. Dermatol 142 (6), 1703–1713. 10.1016/j.jid.2021.09.033 34756879

[B112] Yang C. Xiang H. Fu K. Jin L. Yuan F. Xue B. (2022). Lycorine suppresses cell growth and invasion via down-regulation of NEDD4 ligase in bladder cancer. Am. J. Cancer Res. 12 (10), 4708–4720. 36381314 PMC9641406

[B113] Yao Q. Liu J. Xiao L. Wang N. (2019). Sonic hedgehog signaling instigates high-fat diet-induced insulin resistance by targeting PPARγ stability. J. Biol. Chem. 294 (9), 3284–3293. 10.1074/jbc.RA118.004411 30573683 PMC6398147

[B114] Ye W. Li Y. Wang W. Pan D. Wang X. Gu Y. (2026). NEDD4 promotes reactive astrogliosis by enhancing K63-linked ubiquitination and inhibiting chaperone-mediated autophagy degradation of YAP1. Int. J. Biol. Sci. 22 (2), 858–875. 10.7150/ijbs.123639 41522361 PMC12781170

[B115] Yuen E. Y. Wei J. Liu W. Zhong P. Li X. Yan Z. (2012). Repeated stress causes cognitive impairment by suppressing glutamate receptor expression and function in prefrontal cortex. Neuron 73 (5), 962–977. 10.1016/j.neuron.2011.12.033 22405206 PMC3302010

[B116] Zaro B. W. Yang Y. Y. Hang H. C. Pratt M. R. (2011). Chemical reporters for fluorescent detection and identification of O-GlcNAc-modified proteins reveal glycosylation of the ubiquitin ligase NEDD4-1. Proc. Natl. Acad. Sci. U. S. A. 108 (20), 8146–8151. 10.1073/pnas.1102458108 21540332 PMC3100932

[B117] Zhang H. Nie W. Zhang X. Zhang G. Li Z. Wu H. (2013). NEDD4-1 regulates migration and invasion of glioma cells through CNrasGEF ubiquitination *in vitro* . PLoS One 8 (12), e82789. 10.1371/journal.pone.0082789 24340059 PMC3858320

[B118] Zhang J. Li X. Zhang Y. (2017). Correlation of NEDD4-1 and PTEN expression with the invasive capacity of pituitary adenomas. Mol. Clin. Oncol. 6 (1), 96–100. 10.3892/mco.2016.1092 28123738 PMC5244870

[B119] Zhang W. Yang X. Chen L. Liu Y. Y. Venkatarangan V. Reist L. (2021). A conserved ubiquitin- and ESCRT-dependent pathway internalizes human lysosomal membrane proteins for degradation. PLoS Biol. 19 (7), e3001361. 10.1371/journal.pbio.3001361 34297722 PMC8337054

[B120] Zhong S. X. Liu Z. H. (2009). Expression patterns of Nedd4 isoforms and SGK1 in the rat cochlea. Acta Otolaryngol. 129 (9), 935–939. 10.1080/00016480802552501 19051070

[B121] Zhong S. X. Hu G. H. Liu Z. H. (2014). Expression of ENaC, SGK1 and Nedd4 isoforms in the cochlea of guinea pig. Folia Histochem. Cytobiol. 52 (2), 144–148. 10.5603/FHC.2014.0010 25007182

[B122] Zhou Y. Qiu J. Liu S. Wang P. Ma D. Zhang G. (2023). CFDP1 promotes hepatocellular carcinoma progression through activating NEDD4/PTEN/PI3K/AKT signaling pathway. Cancer Med. 12 (1), 425–444. 10.1002/cam4.4919 35861040 PMC9844661

[B123] Zhu Y. Lei L. Wang X. Chen L. Li W. Li J. (2023). The E3 ubiquitin ligase NEDD4-1 protects against acetaminophen-induced liver injury by targeting VDAC1 for degradation. Acta Pharm. Sin. B 13 (4), 1616–1630. 10.1016/j.apsb.2023.01.019 37139424 PMC10150139

